# DDAH1 Promotes Cisplatin Chemoresistance in Patients with Locally Advanced Nasopharyngeal Carcinoma via the EGFR‐JAK2‐STAT3 Pathway

**DOI:** 10.1002/advs.202503647

**Published:** 2025-06-19

**Authors:** Jin‐Hao Yang, Li Yuan, Qiu‐Yan Chen, Kai‐Qi Lan, Liang‐Ji Li, Yu‐Chen Li, Xiao‐Yun Li, Xue‐Song Sun, Lin‐Quan Tang, Sai‐Lan Liu, Hai‐Qiang Mai

**Affiliations:** ^1^ State Key Laboratory of Oncology in South China Collaborative Innovation Center for Cancer Medicine Guangdong Key Laboratory of Nasopharyngeal Carcinoma Diagnosis and Therapy Sun Yat‐sen University Cancer Center Guangzhou Guangdong 510060 P. R. China; ^2^ Department of Nasopharyngeal Carcinoma Sun Yat‐sen University Cancer Center Guangzhou Guangdong 510060 P. R. China

**Keywords:** cisplatin resistance, DDAH1, EGFR, JAK2‐STAT3 pathway, nasopharyngeal carcinoma, nimotuzumab

## Abstract

Cisplatin‐based induction chemotherapy (IC) improves survival in patients with locally advanced nasopharyngeal carcinoma (LANPC). However, ≈30% of patients with LANPC receiving IC develop chemoresistance, and 20% experience disease progression. The relation between chemoresistance and Dimethylarginine dimethylaminohydrolase‐1 (DDAH1) in NPC has not been mentioned in previous studies. To explore the regulatory mechanism and biological function of DDAH1 in cisplatin chemoresistance, NPC cell lines are subjected to overexpression and knockdown of DDAH1 in vitro, with findings further corroborated by in vivo chemosensitivity assays. The predictive value of DDAH1 expression is evaluated for survival and resistance to cisplatin‐based IC in a cohort of 339 patients with LANPC. Overexpression of DDAH1 in NPC cell lines increases cisplatin resistance both in vitro and in vivo through binding to the intracellular domain of epidermal growth factor receptor (EGFR), enhancing its dimerization and phosphorylation, thereby promoting the activation of the JAK2‐STAT3 pathway, which is dependent on EGFR and extracellular ligands and can be weakened by nimotuzumab. Clinically, DDAH1 positivity correlates with unfavorable 3‐year survivals. This study identified DDAH1 as a prognostic marker and a potential therapeutic target for nimotuzumab to overcome treatment failure and chemoresistance in LANPC and other EGFR‐positive cancers.

## Graphical abstract

This study identifies a novel mechanism of nasopharyngeal carcinoma (NPC) cisplatin chemoresistance that DDAH1 expression is associated with cisplatin resistance in NPC cells primarily through the enhancement of the phosphoinositide Janus kinase 2 (JAK2)‐signal transducer and activator of transcription 3 (STAT3) pathway. Notably, this is the first study to report the interaction between DDAH1 and epidermal growth factor receptor (EGFR). This study demonstrated that this interaction increases the dimerization and phosphorylation of EGFR, which enhances the activation of JAK2‐STAT3 signaling pathway, contributing to increasement of cisplatin resistance in NPC cells. More importantly, nimotuzumab can weakened the cisplatin resistance effect of DDAH1, which provides a new perspective to the use of nimotuzumab.

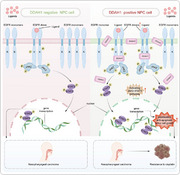

## 1 Introduction

Nasopharyngeal carcinoma (NPC) is an epithelial tumor of the nasopharynx closely associated with Epstein‐Barr virus (EBV) infection.^[^ ^1^ ^]^ It is also characterized by a global geographical distribution,^[^ ^1^ ^]^ with particularly high prevalence among young and middle‐aged individuals in East and Southeast Asia which imposes a considerable burden on patients' families.^[^ ^2^, ^3^ ^]^ Moreover, NPC differs from other epithelial head and neck cancers because it does not originate from the same cell or tissue lineages.^[^ ^2^ ^]^

Concurrent chemoradiotherapy (CCRT) is the standard treatment for patients with locally advanced NPC (LANPC),^[^ ^2^, ^4^, ^5^ ^]^ and the addition of cisplatin‐based induction chemotherapy (IC) is considered to prolong survival in LANPC. However, ≈30% of patient with LANPC are resistant to IC and an unsatisfactory tumor response to IC is considered an adverse prognostic factor in these patients.^[^ ^6^, ^7^ ^]^ Previous studies showed that ≈20% of patients with LANPC who received IC plus CCRT experienced disease recurrence and death due to chemoresistance.^[^ ^5^, ^8^ ^]^ The misregulation of tumor‐related pathways, including various genes, proteins, and peptide products such as epithelial‐mesenchymal transition, cancer stem cells, and the tumor microenvironment, plays an important role in chemoresistance development.^[^ ^9^ ^]^ Additional mechanisms contributing to LANPC chemoresistance include EBV‐related factors, abnormal DNA damage repair, and ATP‐binding cassette transporters.^[^ ^9^ ^]^ However, these studies did not lead to effective treatment methods to solve drug resistance. As IC regimen for LANPC, such as GP regimen (gemcitabine and cisplatin), TP regimen (docetaxel and cisplatin) and TPC (docetaxel, cisplatin and capecitabine) regimen are all based on cisplatin, besides, NCCN guidelines states that cisplatin‐based chemotherapy is of great importance in NPC treatments,^[^ ^6^, ^10^ ^]^ it is crucial to identify novel molecular markers of cisplatin resistance in patients with LANPC, as a better understanding of these mechanisms may help to provide innovative strategies to stratify precise risk and guide individual treatment in patients with LANPC and chemoresistant. Here, using gene regulatory network analysis, we identify Dimethylarginine dimethylaminohydrolase‑1 (DDAH1), a cysteine hydrolase enzyme, as a specific gene that is consistently upregulated in patients with LANPC and chemoresistant. Dimethylarginine dimethylaminohydrolase regulates the cellular concentration of methylarginines, which inhibit nitric oxide synthase (NOS).^[^ ^11^ ^]^ DDAH1 degrades asymmetric dimethylarginine (ADMA), an endogenous NOS inhibitor,^[^ ^11^ ^]^ thereby potentially enhancing NO production. Recently, high expression of DDAH1 has been linked to poor clinical outcomes in certain cancers, such as breast cancer, melanoma, and hepatocellular carcinoma.^[^ ^12^, ^13^, ^14^ ^]^ However, the role of DDAH1 in cisplatin resistance in patients with LANPC remains largely unknown. A previous study suggested that DDAH1 expression is associated with poor survival in patients with LANPC, positioning it as a novel therapeutic target for chemotherapy‐resistant patients.^[^ ^15^ ^]^

Janus kinase 2 (JAK2)‐signal transducer and activator of transcription 3 (STAT3) pathway is a crucial intracellular signaling cascade pathway.^[^ ^16^, ^17^ ^]^ It transmits extracellular signals of cytokines and growth factors to the cell nucleus, where it regulates gene expression and controls the growth of various cells. The activation of the JAK2‐STAT3 pathway starts with the binding of cytokines to receptors, which activates JAK2, and subsequently leads to the phosphorylation of STAT3. The phosphorylated STAT3 enters the cell nucleus in the form of a dimer and regulates the expression of target genes, which promotes cell growth and causes immune escape.^[^ ^17^ ^]^ As for the relation between JAK2‐STAT3 and cancers, several studies reported that abnormal activation of JAK2‐STAT3 pathway results in disease progression and poor survivals of patients with colon and gastric cancers.^[^ ^18^, ^19^ ^]^

In this study, we demonstrated that DDAH1 interacts with EGFR, promoting EGFR dimerization and autophosphorylation, thereby activating the downstream JAK2‐STAT3 signaling pathway and ultimately inducing cisplatin resistance in NPC cells. Our findings identifiy DDAH1as a novel potential therapeutic target for overcoming cisplatin resistance in LANPC.

## 2 Results

### 2.1 DDAH1 is Associated with Cisplatin Resistance and Poor Prognosis in Patients with LANPC

**Figure 1:**
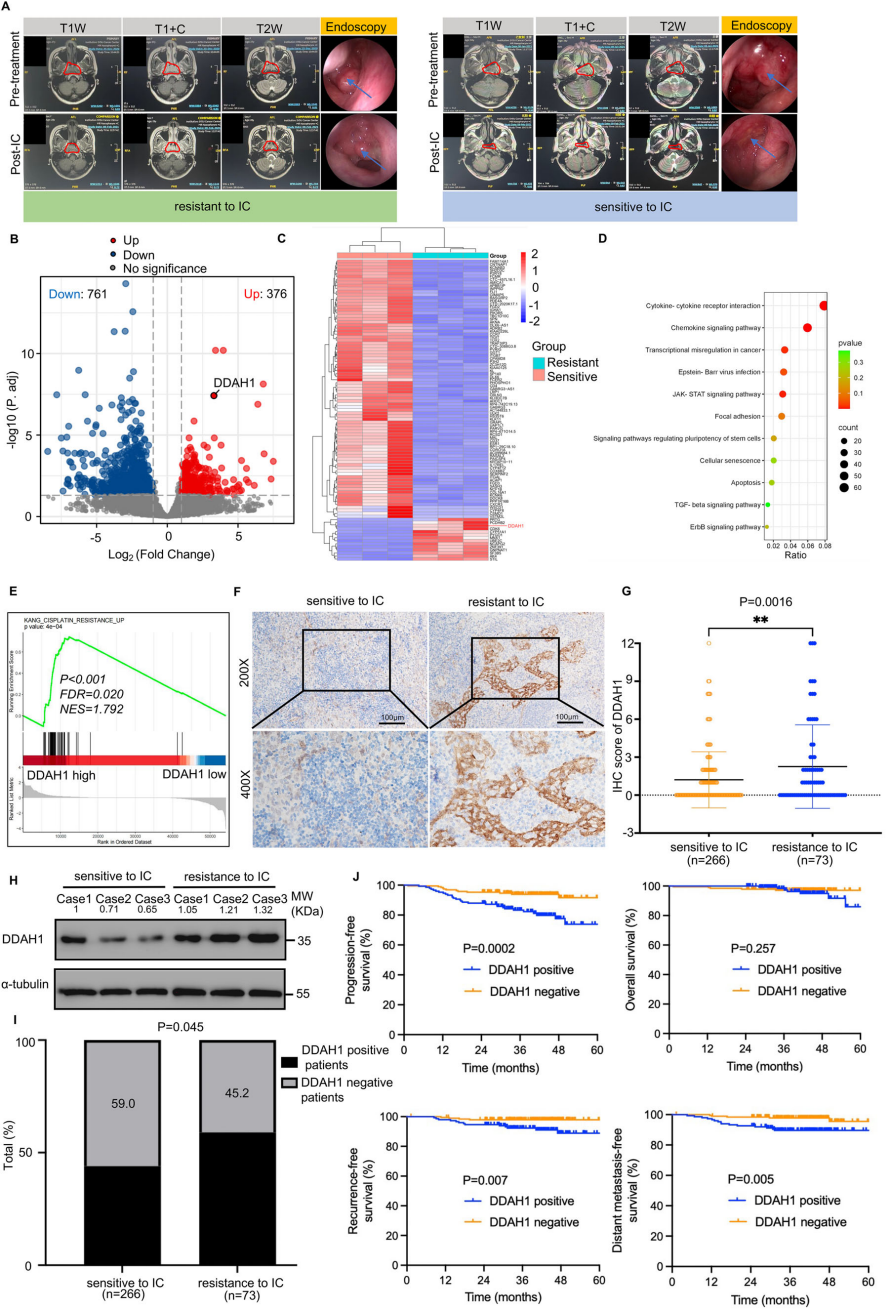
DDAH1 is associated with cisplatin resistance and poor prognosis in patients with locally advanced nasopharyngeal carcinoma (LANPC). A), Typical magnetic resonance imaging (MRI) photos of pretreatment and post‐IC of patients with LANPC resistant and sensitive to induction chemotherapy (IC). “T1W” is abbreviation of “T1‐weighted image”; “T1+C” is an abbreviation of “T1‐weighted contrast‐enhanced image”; “T2W” is abbreviation of “T2‐weighted image”. B, C), Volcano plot and heatmap show that DDAH1 is one of the upregulated genes by comparing three patients with LANPC resistant to IC with the three patients sensitive to IC. D), Bubble diagram of Kyoto Encyclopedia of Genes and Genomes (KEGG) analysis of differentially expressed genes reveals tumor‐related pathways. E), Gene set enrichment (GSEA) analysis indicates that cisplatin resistance is affected by DDAH1 expression. F), Immunohistochemical (IHC) characteristics of DDAH1 in patients with LANPC resistance and sensitive to IC. Scale bar, 100 µm. G), IHC score of DDAH1 in patients with LANPC resistant and sensitive to IC. Significance is calculated using unpaired two‐tailed Student's t‐test, ^**^ *p* < 0.01. H), Western blot analyses show the expression of DDAH1 in tissue samples of three patients with LANPC sensitive to IC and three patients resistant to IC. I), Comparison of the percentage of patients that are DDAH1‐negative or ‐positive in patients with LANPC sensitive and resistant to IC. P value is calculated by chi‐square test. J), 3‐year progression‐free survival (PFS), 3‐year overall survival (OS), 3‐year recurrence‐free survival (RFS) and 3‐year distant metastasis‐free survival (DMFS) for DDAH1‐positive and ‐negative groups of the 339 patients with LANPC. P values are calculated by log‐rank test.

In our study, 339 patients received cisplatin‐based IC plus CCRT. Among them, 266 patients were sensitive to IC (imaging evaluation showed complete response or partial response after IC), and 73 patients were resistant to IC (imaging evaluation showed stable disease or progressive disease after IC). **Figure** **1**A presents typical magnetic resonance imaging images of patients with LANPC who were resistant and sensitive to IC. To identify key factors affecting chemoresistance, we selected pretreated NPC tissues from three patients with LANPC resistant to IC and three patients sensitive to IC for whole‐transcriptome analysis. This analysis revealed that DDAH1 expression was upregulated in IC‐resistant patients (Figure 1B,C). KEGG analysis suggested that the differentially expressed genes were enriched in tumor‐related pathways (Figure 1D). GSEA demonstrated that DDAH1 affects cisplatin resistance (Figure 1E). Next, immunohistochemistry (IHC) was performed to examine DDAH1 expression in LANPC tissues from the 339 patients with LANPC. The results showed that DDAH1 expression was higher in patients with IC‐resistant than in patients with IC‐sensitive (Figure 1F,G), however, DDAH1 does not specifically highly express in NPC tissues compared with normal nasopharyngeal tissues (Figure S2, Supporting Information). Western blot also revealed that DDAH1 was upregulated in the tissues of patients with IC‐resistant, and DDAH1‐positive cells were associated with IC resistance (Figure 1H,I). Furthermore, the 3‐year progression‐free survival (PFS), recurrence‐free survival (RFS), and distant metastasis‐free survival (DMFS) of patients with LANPC and DDAH1‐negative were better than those of patients with DDAH1‐positive. However, there was no significant difference in 3‐year overall survival (OS) between patients with DDAH1‐positive and DDAH1‐negative (Figure 1J). Further analysis identified DDAH1 positivity as an independent prognostic biomarker in patients with LANPC (Table S6, Supporting Information).

### 2.2 DDAH1 is Associated with Cisplatin Resistance In Vitro

**Figure 2:**
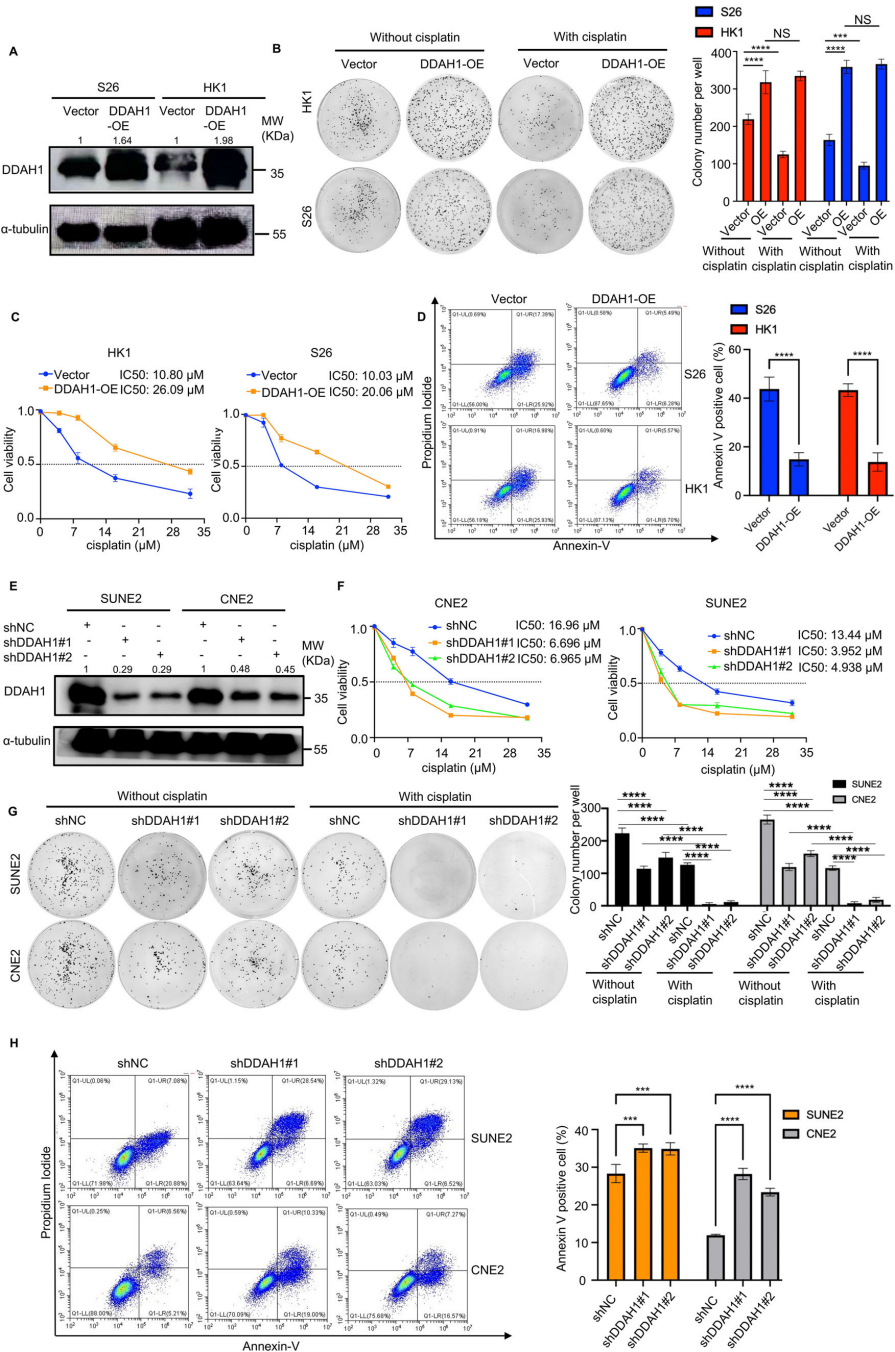
DDAH1 is associated with cisplatin resistance in NPC cell lines. A), Western blot analyses show the expression of DDAH1 in S26 and HK1 NPC cell lines with DDAH1 overexpression plasmid and empty vector plasmid as control. B), Quantified results of colony formation assays in S26 and HK1 cell lines with DDAH1 overexpression and empty vector plasmids treated with cisplatin at 4 µm for 36 h and PBS as control. The data are presented as mean ± SD and are representative of three independent experiments. Significances are calculated by one‐way ANOVA with Tukey's multiple comparisons. NS, no significance, ^***^ *p* < 0.001, ^****^ *p* < 0.0001. “OE” is the abbreviation for “DDAH1‐OE”. C), Cell Counting Kit 8 (CCK8) analysis for S26 and HK1 cell lines expressing DDAH1 overexpression plasmid (and empty vector) after treatment with cisplatin at the indicated concentrations (0, 4, 8, 16, and 32 µm) for 36 h. The data are presented as mean ± SD and are representative of three independent experiments. D), Flow cytometry analysis for S26 and HK1 cell lines expressing DDAH1 overexpression plasmid (and empty vector) after treatment with cisplatin at 8 µm for 36 h. The data are presented as mean ± SD and are representative of three independent experiments. Significances are calculated using unpaired two‐tailed Student's t‐test, ^****^ *p* < 0.0001. E), Western blot analyses show the expression of DDAH1 in SUNE2 and CNE2 cell lines with DDAH1 knockdown and shNC as control. F), CCK8 analysis for SUNE2 and CNE2 cell lines with DDAH1 knockdown (and shNC as control) after treatment with cisplatin at the indicated concentrations (0, 4, 8, 16, and 32 µm) for 36 h. The data are presented as mean ± SD and are representative of three independent experiments. G), Quantified results of colony formation assays in CNE2 and SUNE2 cell lines with DDAH1 knockdown and shNC treated with cisplatin at 6 µm for 36 h and PBS as control. The data are presented as mean ± SD and are representative of three independent experiments. Significances are calculated by one‐way ANOVA with Tukey's multiple comparisons. ^****^ *p* < 0.0001. H, Flow cytometry analysis for SUNE2 and CNE2 cell lines with DDAH1 knockdown and shNC after treatment with cisplatin at 6 µm for 36 h. The data are presented as mean ± SD and are representative of three independent experiments. Significances are calculated by one‐way ANOVA with Tukey's multiple comparisons. ^***^ *p* < 0.001, ^****^ *p* < 0.0001.

Based on the clinical observations of DDAH1 and cisplatin‐based chemotherapy resistance, we selected two NPC cell lines (HK1 and S26) stably expressing the DDAH1 overexpression plasmid and set an empty vector (**Figure** **2**A). We then performed CCK8 analysis and colony formation assays to assess cell viability and used flow cytometry to estimate the percentage of apoptotic cells after adding cisplatin to the culture medium. DDAH1 overexpression enhanced cisplatin resistance in the NPC cell line (Figure 2B, C,D). Additionally, we knocked down DDAH1 in SUNE2 and CNE2 cell lines (Figure 2E), and repeated the above experiments to estimate the percentage of apoptosis after adding cisplatin. As shown in Figure 2F,G,H, the downregulation of DDAH1 expression in SUNE2 and CNE2 cell lines increased their sensitivity to cisplatin.

### 2.3 NPC Cell Line Stemness is Increased by DDAH1 Overexpression

**Figure 3:**
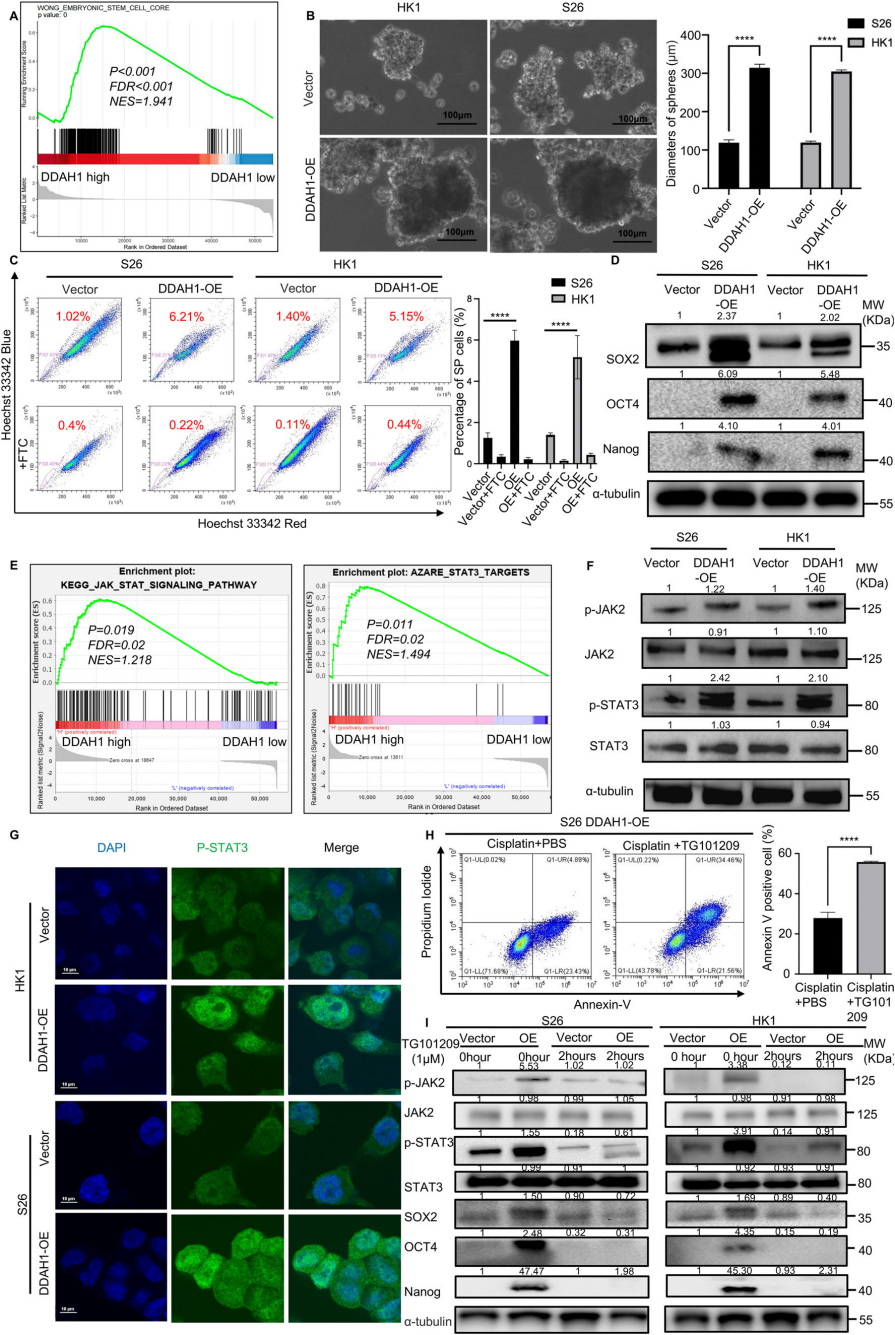
Overexpression of DDAH1 enhances stemness of NPC cell lines and confers resistance to cisplatin by activating the JAK2‐STAT3 pathway. A), DDAH1 affects cell stemness based on GSEA. B), Sphere formation assay of S26 and HK1 cell lines with DDAH1 overexpression plasmid (and empty vector plasmid is set as control). Scale bar, 100 µm. The data are presented as mean ± SD and are representative of 5 independent experiments. Diameters of spheres are shown. Significances are calculated by unpaired two‐tailed Student's t test, ^****^ *p* < 0.0001. C), Side population (SP) cells are detected in S26 and HK1 cell lines with the DDAH1 overexpression plasmid (and empty vector plasmid is set as control). The data are presented as mean ± SD and are representative of three independent experiments. Significances are calculated by one‐way ANOVA with Tukey's multiple comparisons. ^****^ *p* < 0.0001. “OE” is the abbreviation for “DDAH1‐OE”. D), Western blot analyses show the expression of *Sox2*, *Oct4*, and *Nanog* in S26 and HK1 NPC cell lines expressing DDAH1 overexpression plasmid (and empty vector plasmid is set as control). E), GSEA shows that DDAH1 affects JAK‐STAT and STAT3 related pathways in the S26 cell line expressing the DDAH1 overexpression plasmid and empty vector plasmid. F, Western blot analyses show the expression of p‐JAK2, p‐STAT3, JAK2, and STAT3 in S26 and HK1 cell lines expressing DDAH1 overexpression plasmid (and empty vector). G), IF shows the location of p‐STAT3 in S26 and HK1 cell lines expressing DDAH1 overexpression plasmid (and empty vector). Scale bar, 10 µm. H), Flow cytometry analysis for S26 cell line expressing DDAH1 overexpression plasmid treated with cisplatin at 8 µm for 36 h plus PBS for 2 h and cisplatin at 8 µm for 36 h plus TG101209 1 µm for 2 h. The data are presented as mean ± SD and are representative of three independent experiments. Significances are calculated by unpaired two‐tailed Student's t test, ^****^ *p* < 0.0001. I), Western blot analyses show the expression of p‐JAK2, p‐STAT3, JAK2, STAT3, *Sox2*, *Oct4*, and *Nanog* in S26 and HK1 cell lines with DDAH1 overexpression plasmid (and empty vector plasmid is set as control) treated with TG101209 at 1 µm for 0 and 2 h. “OE” used in the figure is an abbreviation for “DDAH1‐OE”.

According to GSEA analysis, DDAH1 affects cancer cell stemness (**Figure** **3**A). The sphere assay showed that larger NPC spheres formed in the DDAH1‐overexpressing HK1 and S26 NPC cell lines compared to the control groups (Figure 3B). Furthermore, we compared side population (SP) cells when DDAH1 was overexpressed in HK1 and S26 cells with the control groups. The proportion of SP cells was 6.21% when DDAH1 was overexpressed, whereas it was 1.02% in the control group of the S26 cell line (Figure 3C). The rate of SP cells was 5.15% when DDAH1 was overexpressed, while it was 1.40% in the control group in the HK1 cell line (Figure 3C). Additionally, western blotting and qPCR showed that DDAH1 overexpression significantly upregulated *Sox2*, *Oct4*, and *Nanog*, which are closely related to cancer cell stemness (Figure 3D; Figure S3, Supporting Information).^[^ ^20^ ^]^

### 2.4 Overexpression of DDAH1 Confers Cisplatin Resistance in NPC Cell Lines Through Enhancement of the JAK2‐STAT3 Pathway

According to GSEA, DDAH1 was found to affect the JAK‐STAT and STAT3‐related signaling pathways (Figure 3E) in S26 cell lines expressing the DDAH1 overexpression plasmid. Western blot analysis indicated that overexpression of DDAH1 led to increased phosphorylation levels of JAK2 (p‐JAK2) and STAT3 (p‐STAT3) (Figure 3F). Additionally, immunofluorescence (IF) analysis showed that DDAH1 overexpression enhanced the translocation of p‐STAT3 to the nucleus (Figure 3G). Next, cisplatin and TG101209 (a JAK2 inhibitor) were added to the S26 cell line with DDAH1 overexpression. Apoptosis in the S26 cells overexpressing DDAH1 was significantly increased when TG101209 was combined with cisplatin, compared to cisplatin treatment alone (Figure 3H). Western blot analysis showed that the addition of TG101209 reduced the expression of p‐JAK2, p‐STAT3, *Oct4*, *Nanog*, and *Sox2* (Figure 3I). Therefore, overexpression of DDAH1 increases the stemness of NPC cell lines and increases their resistance to cisplatin by enhancing the JAK2‐STAT3 pathway.

### 2.5 DDAH1 Interacts with the intracellular domain of EGFR in NPC Cell Lines

**Figure 4:**
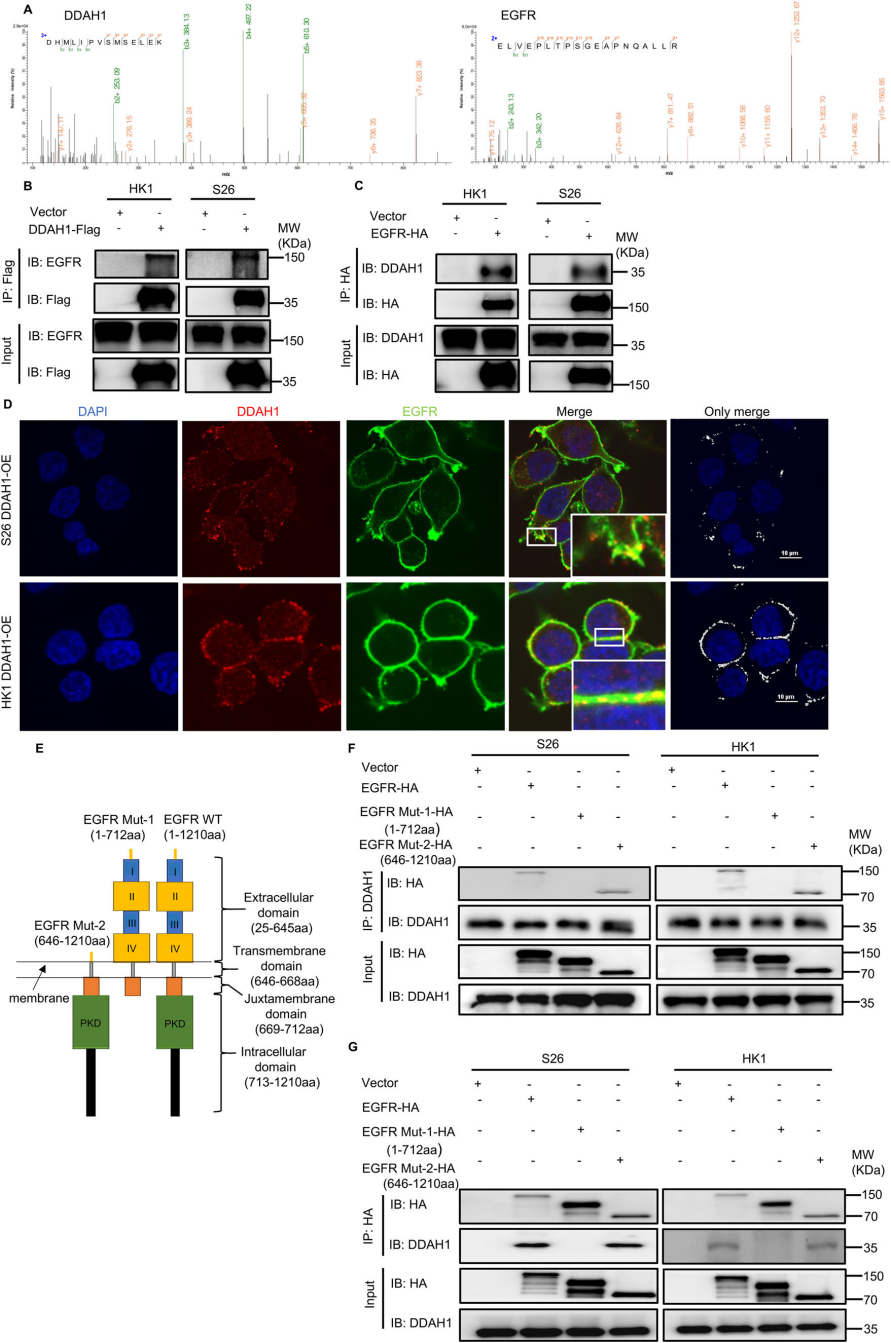
DDAH1 interacts with the intracellular domain of EGFR in NPC cell lines. A), Mass spectrometry (MS) detection shows the peptide plot of DDAH1 and EGFR. B), Lysates from wildtype S26 and HK1 cell lines transfected with plasmids expressing DDAH1‐Flag are immunoprecipitated with an anti‐Flag antibody and subjected to western blot analysis. C), Lysates from wildtype S26 and HK1 cell lines transfected with plasmids expressing EGFR‐HA are immunoprecipitated with an anti‐HA antibody and subjected to western blot analysis. D), IF showing the location and combination of EGFR and DDAH1 in S26 and HK1 cell lines expressing DDAH1 overexpression plasmid. Scale bar, 10 µm. E), Schematic illustration of the EGFR domain deletion construct used in F,G). F, Lysates from wildtype S26 and HK1 NPC cell lines transfected with plasmids expressing EGFR‐HA, EGFR‐Mutation‐1‐HA (EGFR‐Mut‐1‐HA in the figure), and EGFR‐Mutation‐2‐HA (EGFR‐Mut‐2‐HA in the figure) are immunoprecipitated with an anti‐DDAH1 antibody and subjected to western blot analysis. G), Lysates from wildtype S26 and HK1 NPC cell lines transfected with plasmids expressing EGFR‐HA, EGFR‐Mutation‐1‐HA (EGFR‐Mut‐1‐HA in the figure), and EGFR‐Mutation‐2‐HA (EGFR‐Mut‐2‐HA in the figure) are immunoprecipitated with an anti‐HA antibody and subjected to western blot analysis.

To further investigate the mechanism linking DDAH1 to the JAK2‐STAT3 pathway and cisplatin resistance, we carried out DDAH1 overexpression plasmid transfection and pull‐down mass spectrometry to find out the proteins that interact with DDAH1, the result showed that EGFR interacts with DDAH1 (**Figure** **4**A; Figure S4, Supporting Information). To confirm this finding, plasmids containing DDAH1 and FLAG tags, along with plasmids containing EGFR and HA tags, were transfected into both S26 and HK1 wild‐type cell lines. The results showed that DDAH1 interacted with EGFR (Figure 4B,C). Additionally, IF analysis demonstrated that DDAH1 and EGFR were co‐localized in both S26 and HK1 cell lines overexpressing DDAH1 (Figure 4D). To determine the specific regions where DDAH1 and EGFR interact, truncated plasmids were created for the extracellular domain, transmembrane (TM) domain, and juxtamembrane domain of EGFR (1‐712), as well as for the TM domain, juxtamembrane domain, and intracellular domain of EGFR (646‐1210) (Figure 4E) according to previous studies.^[^^]^ These two plasmids were transfected into S26 and HK1 wild‐type cell lines. Our findings revealed that DDAH1 binds to the intracellular domain of EGFR (646‐1210) (Figure 4F,G).

### 2.6 DDAH1 Enhances the JAK2‐STAT3 Pathway by Promoting the Phosphorylation and Dimerization of EGFR in NPC Cell Lines

**Figure 5:**
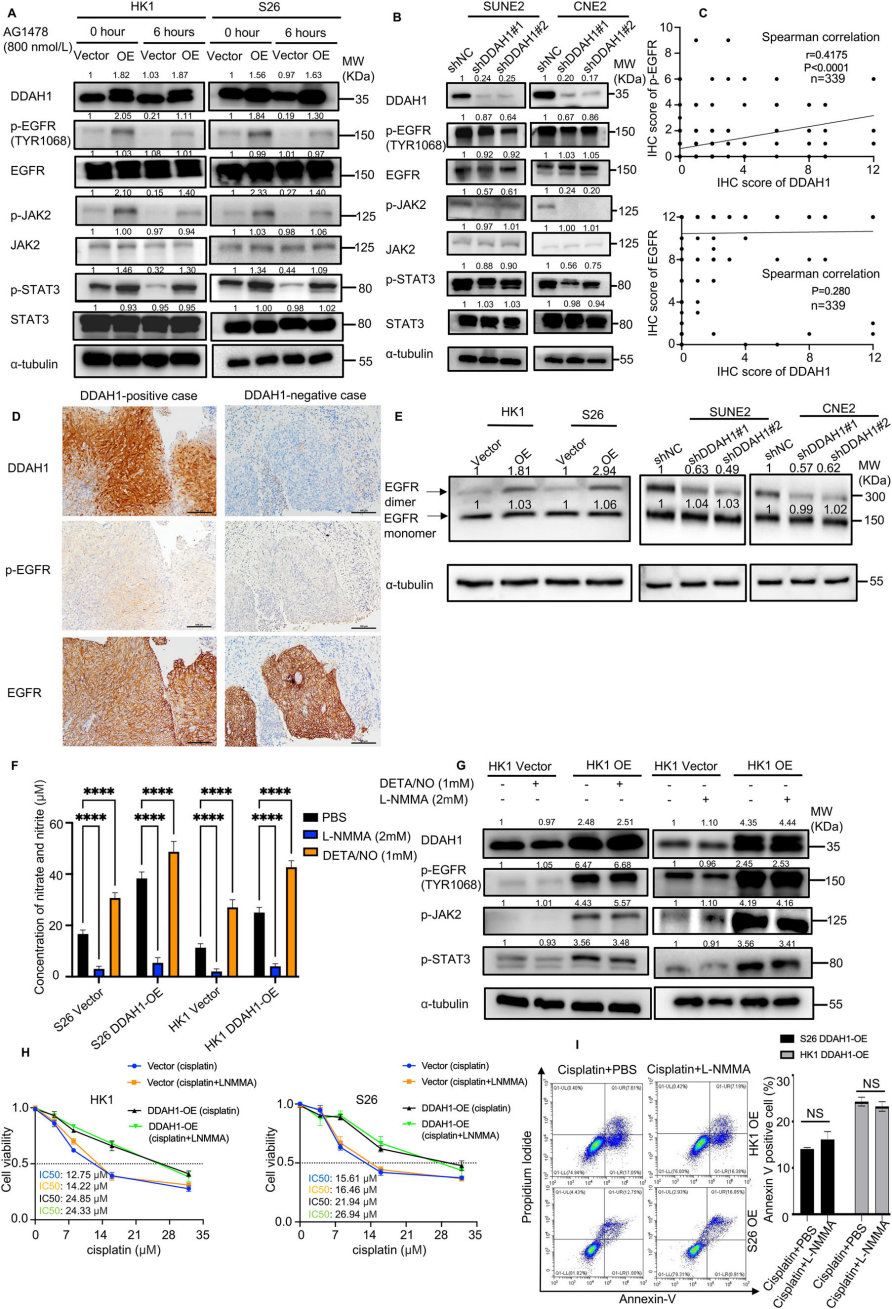
DDAH1 enhances cisplatin resistance and the JAK2‐STAT3 pathway via phosphorylation of EGFR in NPC cell lines independent of intracellular NO concentration. A), Western blot analyses show the expression of DDAH1, p‐EGFR (TYR1068), p‐JAK2, p‐STAT3, JAK2, and STAT3 in S26 and HK1 cell lines expressing the DDAH1 overexpression plasmid (and empty vector) after using AG1478 at 800 nmol L^−1^ for 6 h, and without using AG1478 is set as control. “OE” is an abbreviation for “DDAH1‐OE”. B), Western blot analyses show the expression of DDAH1, p‐EGFR (TYR1068), p‐JAK2, p‐STAT3, JAK2, and STAT3 in SUNE2 and CNE2 cell lines with DDAH1 knockdown and shNC is set as control. C), Spearman correlation analysis for the relation between the expression of DDAH1 and EGFR, and the relation between the expression of DDAH1 and p‐EGFR. D), IHC characteristics of DDAH1, EGFR and p‐EGFR (TYR1068) in LANPC patients with positive DDAH1 and negative DDAH1 expressions. Scale bar, 100 µm. E), Western blot analyses show the expression of EGFR dimer and monomer by cross‐linking in S26 and HK1 cell lines expressing DDAH1 overexpression plasmid (and empty vector), and in SUNE2 and CNE2 cell lines with DDAH1 knockdown and shNC is set as control. “OE” is an abbreviation for “DDAH1‐OE.” The fold changes are for EGFR monomers and dimers and are normalized by α‐tubulin. F), Concentrations of nitrite in S26 and HK1 NPC cell lines expressing DDAH1 overexpression plasmid (and empty vector) in L‐NMMA, DETA/NO and control groups. L‐NMMA is treated at 2 mm for 1 h. DETA/NO is treated at 1 mm for 24 h. The data are presented as mean ± SD and are representative of three independent experiments. Statistical significances are determined by using one‐way ANOVA with Tukey's multiple comparisons. ^****^ *p* < 0.0001. G), Western blot analyses show the expression of p‐EGFR, p‐JAK2, and p‐STAT3 in HK1 NPC cell lines expressing DDAH1 overexpression plasmid (and empty vector) treated without DETA/NO or with DETA/NO at 1 mm for 24 h, and without L‐NMMA or with L‐NMMA at 2 mm for 1 h. “OE” is an abbreviation for “DDAH1‐OE”. H), CCK8 analysis in HK1 and S26 cell lines expressing DDAH1 overexpression plasmid (and empty vector) in cisplatin plus PBS and cisplatin plus L‐NMMA, cisplatin is treated at the indicated concentrations (0, 4, 8, 16, and 32 µm) for 36 h, L‐NMMA is treated at 2 mm for 1 h. The data are presented as mean ± SD and are representative of three independent experiments. I), Flow cytometry analysis for HK1 and S26 cell lines expressing DDAH1 overexpression plasmid after treatment with cisplatin plus PBS and cisplatin plus L‐NMMA. Cisplatin is treated at 8 µm for 36 h. L‐NMMA is treated at 2 mm for 1 h. “OE” is an abbreviation for “DDAH1‐OE.” The data are presented as mean ± SD and are representative of three independent experiments. Statistical significances are determined by using unpaired two‐tailed Student's t test. NS, no significance.

Western blot showed that in both S26 and HK1 cell lines, overexpression of DDAH1 resulted in increased phosphorylation of EGFR (p‐EGFR) (TYR1068), p‐JAK2, and p‐STAT3, whereas knock down of DDAH1 led to decreased phosphorylation of these proteins (Figure **5**A,B). To clarify the role of p‐EGFR in the JAK2‐STAT3 pathway, we used an EGFR‐specific tyrosine kinase inhibitor (AG1478) and observed a significant decrease in the levels of p‐EGFR (TYR1068), p‐JAK2, and p‐STAT3 (Figure 5A). IHC and Spearman's rank correlation analysis showed that the expression of p‐EGFR (TYR1068) was positively correlated with DDAH1 expression in human NPC tissues, however, no significant correlation was found between EGFR and DDAH1 (Figure 5C,D; Figure S5, Supporting Information). These findings suggest that p‐EGFR plays a critical role in the JAK2‐STAT3 pathway. Cross‐linking experiments showed that EGFR dimerization increased when DDAH1 was overexpressed, while dimerization decreased when DDAH1 was knocked down (Figure 5E). These results suggest that DDAH1 enhances EGFR dimerization and the EGFR‐JAK2‐STAT3 pathway.

### 2.7 DDAH1 Enhances the EGFR‐JAK2‐STAT3 Pathway and Cisplatin Resistance Independent of Intracellular NO Concentration and its Enzyme Activity

**Figure 6:**
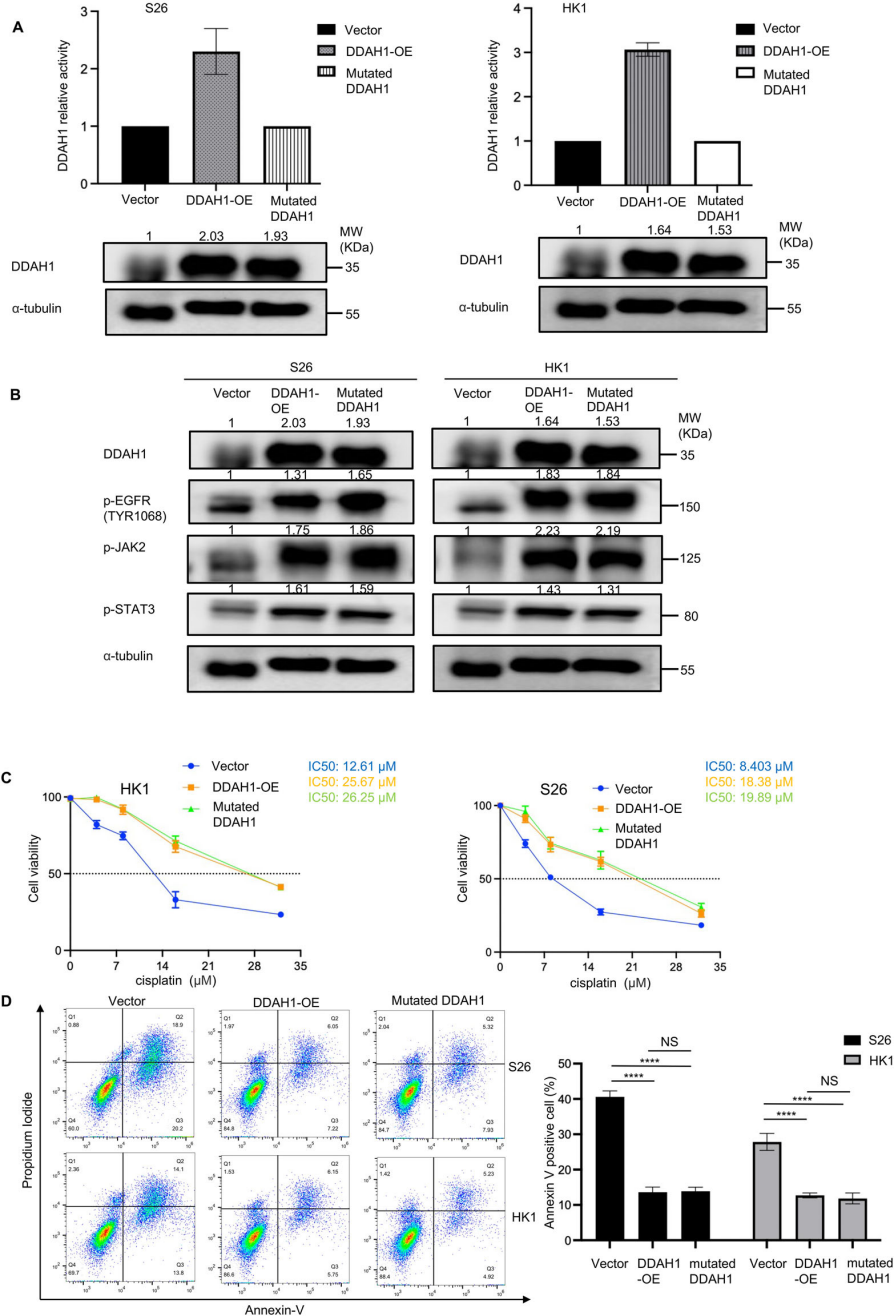
DDAH1 enhances cisplatin resistance and the JAK2‐STAT3 pathway via phosphorylation of EGFR in NPC cell lines independent of DDAH1 enzyme activity. A), DDAH1 relative activity of DDAH1 overexpression and mutated DDAH1 for S26 and HK1 NPC cell lines, vector is set for control. Western blot analyses show the expression of DDAH1 in S26 and HK1 NPC cell lines. B), Western blot analyses show the expression of p‐EGFR (TRY1068), p‐JAK2, p‐STAT3 in S26 and HK1 NPC cell lines with vector, DDAH1 overexpression and mutated DDAH1. C), CCK8 analysis for S26 and HK1 cell lines with vector, DDAH1 overexpression and mutated DDAH1 after treatment with cisplatin at the indicated concentrations (0, 4, 8, 16, and 32 µm) for 36 h. The data are presented as mean ± SD and are representative of three independent experiments. D), Flow cytometry analysis for S26 and HK1 cell lines with vector, DDAH1 overexpression and mutated DDAH1 after treatment with cisplatin at 8 µm for 36 h. The data are presented as mean ± SD and are representative of three independent experiments. Significances are calculated by one‐way ANOVA with Tukey's multiple comparisons. NS, no significance, ^****^ *p* < 0.0001.

DDAH1 plays a crucial role in NO homeostasis.^[^ ^11^, ^24^, ^25^ ^]^ To investigate whether DDAH1 enhances the EGFR‐JAK2‐STAT3 pathway by altering the concentration of NO, DETA/NO (an NO donor) and the endothelial NOS inhibitor *^N^* G‐monomethyl‐L‐arginine (L‐NMMA) was used to substantially increase and decrease intracellular NO concentrations (Figure 5F). Western blot analysis showed that the phosphorylation levels of EGFR (p‐EGFR [TYR1068]), JAK2 (p‐JAK2), and STAT3 (p‐STAT3) did not change significantly in HK1 and S26 cell lines expressing the DDAH1 overexpression plasmid (and empty vector controls) after modulating intracellular NO concentration with DETA/NO or L‐NMMA (Figure 5G; Figure S6A, Supporting Information A). The colony formation assay indicated that L‐NMMA did not affect the sensitivity of S26 and HK1 cell lines to cisplatin (Figure S6B, Supporting Information). Furthermore, CCK8 analysis and flow cytometry showed no significant change in cisplatin sensitivity in HK1 and S26 cell lines expressing DDAH1 overexpression plasmid when the NO concentration was altered (Figure 5H,I). These results suggest that DDAH1 enhances the EGFR‐JAK2‐STAT3 pathway without depending on altering the intracellular NO concentration. Besides, to investigate whether DDAH1 enhances the EGFR‐JAK2‐STAT3 pathway by its enzyme activity, we created a DDAH1 Cys273 to Ser site mutation according to previous studies.^[^^]^ This DDAH1^C273S^ mutant lacks DDAH1 enzyme activity (**Figure** **6**A). Western blot analysis showed that the phosphorylation levels of EGFR (p‐EGFR [TYR1068]), JAK2 (p‐JAK2), and STAT3 (p‐STAT3) did not change significantly in HK1 and S26 cell lines when DDAH1 lacks its enzyme activity (Figure 6B). Furthermore, CCK8 analysis and flow cytometry showed no significant change in cisplatin sensitivity in HK1 and S26 cell lines when DDAH1 lacks its enzyme activity (Figure 6C,D). We also added a DDAH1 inhibitor (PD404182) to inhibit DDAH1 enzyme activity (Figure S7A, Supporting Information), western blot analyses showed that DDAH1 can still enhance EGFR‐JAK2‐STAT3 pathway (Figure S7B, Supporting Information) even if DDAH1 enzyme activity is inhibited. CCK8 analysis and flow cytometry showed no significant change in cisplatin sensitivity in HK1 and S26 cell lines when PD404182 was added to cisplatin (Figure S7C,D, Supporting Information). Therefore, these results suggested that DDAH1 enhances the EGFR‐JAK2‐STAT3 pathway and cisplatin resistance without depending on its enzyme activity.

### 2.8 DDAH1 Enhances the EGFR‐JAK2‐STAT3 Pathway through the Combination of EGFR and Extracellular Ligands

**Figure 7:**
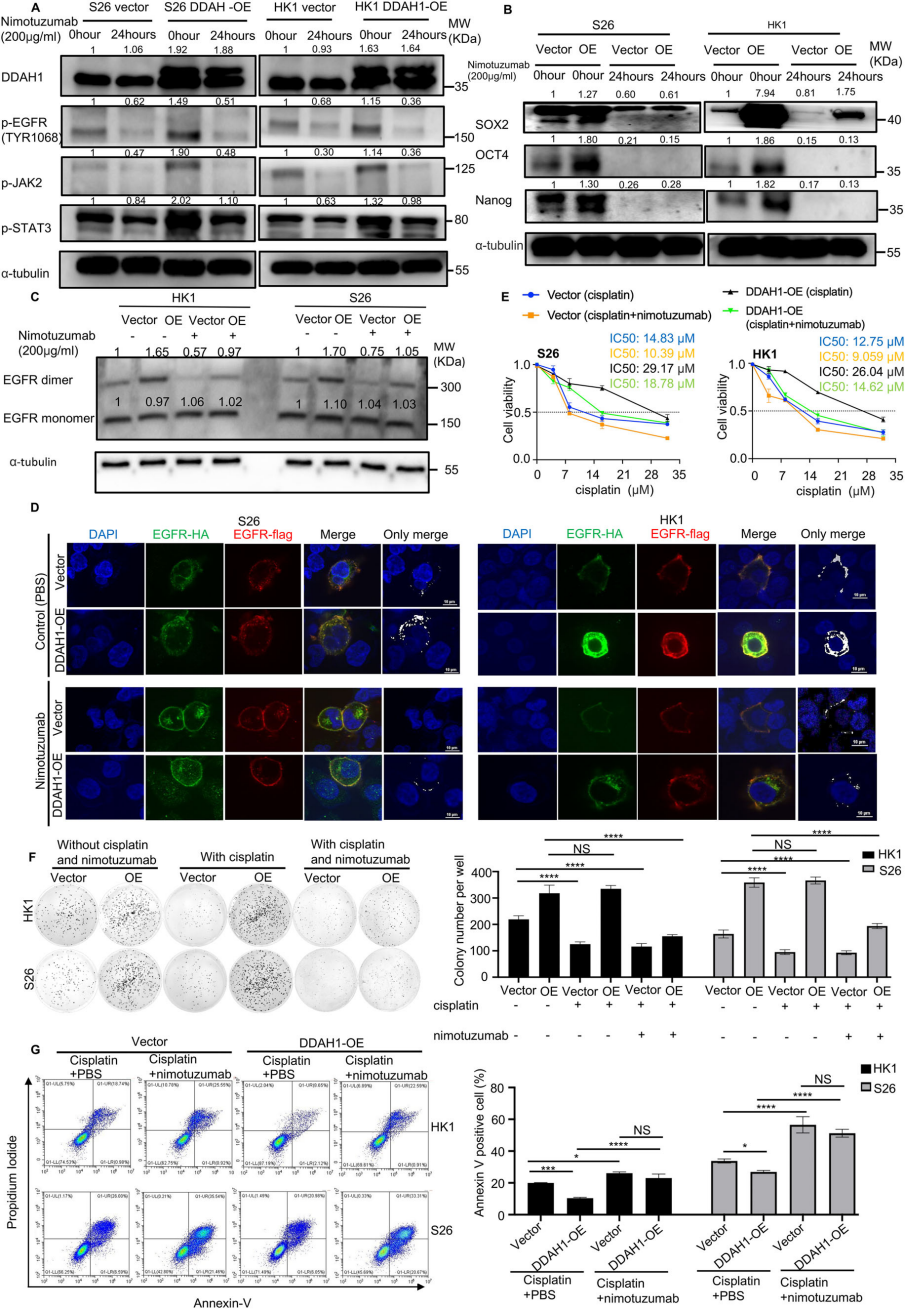
DDAH1 enhances EGFR‐JAK2‐STAT3 pathways through the combination of EGFR and extracellular ligands. A), Western blot analyses show the expression of p‐EGFR, p‐JAK2, and p‐STAT3 in HK1 and S26 cell lines expressing DDAH1 overexpression plasmid (and empty vector) B), Western blot analyses show the expression of *Sox2*, *Oct4*, and *Nanog* in S26 and HK1 cell lines expressing DDAH1 overexpression plasmid (and empty vector). C), Western blot analyses show the expression of EGFR dimer and monomer in HK1 and S26 cell lines expressing DDAH1 overexpression plasmid (and empty vector) treated without nimotuzumab or with nimotuzumab at 200 µg mL^−1^ for 24 h. D), IF shows the combination and location of EGFR‐HA and EGFR‐Flag in HK1 and S26 cell lines expressing DDAH1 overexpression plasmid (and empty vector) treated with nimotuzumab for 0 h and nimotuzumab at 200 µg mL^−1^ for 24 h. Scale bar, 10 µm. E), CCK8 analysis in HK1 and S26 cell lines expressing DDAH1 overexpression plasmid (and empty vector), cisplatin is treated at the different concentrations m for 36 h, nimotuzumab is treated at 200 µg mL^−1^ for 36 h. F), Quantified results of colony formation assays in HK1 and S26 cell lines with DDAH1 overexpression plasmid (and empty vector plasmid is set as control) treated with PBS alone and cisplatin at 6 µm plus PBS for 36 h and cisplatin at 6 µm plus nimotuzumab at 200 µg mL^−1^ for 36 h. G), Flow cytometry analysis for HK1 and S26 NPC cell lines expressing DDAH1 overexpression plasmid (and empty vector). Cisplatin is treated at 8 µm for 36 h. Nimotuzumab is treated at 200 µg mL^−1^ for 36 h. "OE" is abbreviation of “DDAH1‐OE”. E), F), G) The data are presented as mean ± SD and are representative of three independent experiments. F), G) Statistical significances are determined by using one‐way ANOVA with Tukey's multiple comparisons. NS, no significance, ^*^ *p* < 0.05, ^***^ *p* < 0.001, ^****^ *p* < 0.0001.

Activation of EGFR‐related pathways primarily depends on the interaction of EGFR with extracellular ligands.^[^^]^ To determine whether DDAH1's enhancement of the EGFR‐JAK2‐STAT3 pathway depends on the combination of EGFR and extracellular ligands, S26 and HK1 cell lines expressing either the DDAH1 overexpression plasmid or empty vector were treated with nimotuzumab at a concentration of 200 µg mL^−1^ for 24 h to block the binding of extracellular ligands to EGFR. Western blot analysis showed that the phosphorylation levels of EGFR (p‐EGFR [TYR1068]), JAK2 (p‐JAK2), and STAT3 (p‐STAT3), as well as the expression levels of *Sox2*, *Oct4*, and *Nanog* were significantly reduced even with DDAH1 overexpression in S26 and HK1 cell lines (**Figure** **7**A,B). Additionally, nimotuzumab treatment decreased EGFR dimerization in DDAH1‐overexpressing cells (Figure 7C). The existence of EGFR dimerization is shown in Figure 7D. Furthermore, CCK8 analysis, colony formation assays, and flow cytometry showed that nimotuzumab treatment made S26 and HK1 cell lines with DDAH1 overexpression more sensitive to cisplatin than those without nimotuzumab treatment, suggesting that DDAH1 enhances the EGFR‐JAK2‐STAT3 pathway through the interaction of EGFR with extracellular ligands (Figure 7E–G). Besides, when we added PD404182 to inhibit DDAH1 enzyme activity (Figure S7A, Supporting Information), CCK8 analysis and flow cytometry showed that the addition of PD404182 to cisplatin and nimotuzumab is not able to make S26 and HK1 cell lines more sensitive to cisplatin (Figure S7C,D, Supporting Information), which also suggested that DDAH1 enhances the EGFR‐JAK2‐STAT3 pathway and cisplatin resistance through the interaction of EGFR with extracellular ligands but not through DDAH1 enzyme activity.

### 2.9 DDAH1‐Mediated Cisplatin Resistance can be Reversed by Nimotuzumab In Vivo

**Figure 8:**
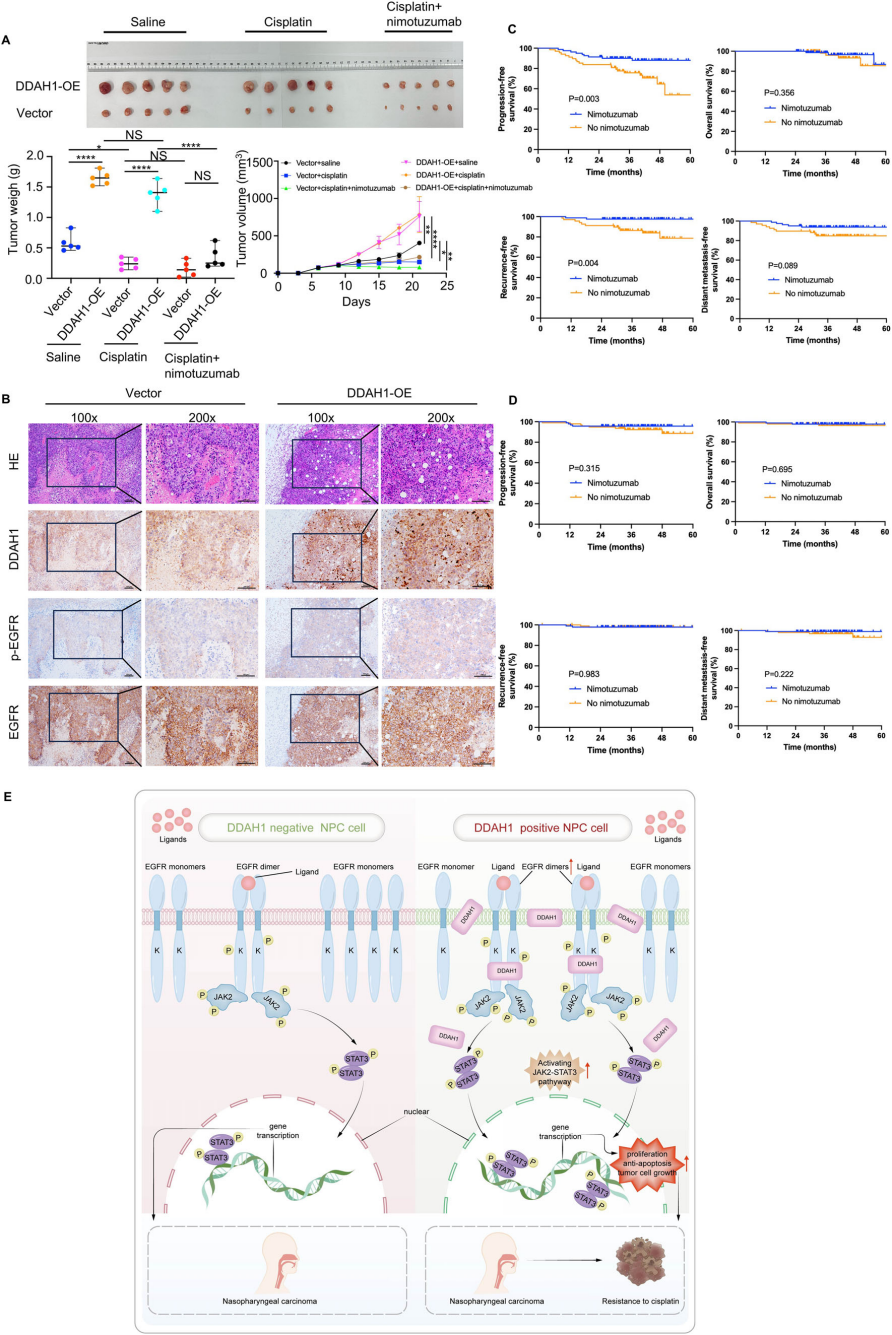
Nimotuzumab decreases the enhancement of cisplatin resistance caused by DDAH1 in vivo and DDAH1 serves as a potential marker for the use of nimotuzumab. A), Subcutaneous injection of the S26 cell line expressing DDAH1 overexpression plasmid (empty vector plasmid is set as control) into M‐NSG mice to establish a mouse model (*n* = 5) treated with saline, cisplatin (4 mg kg^−1^) plus saline, and cisplatin (4 mg kg^−1^) plus nimotuzumab (20 mg kg^−1^). Tumor images, tumor weights, and tumor volumes are shown. Statistical significances are determined by using one‐way ANOVA with Tukey's multiple comparisons. NS, no significance, ^*^ *p* < 0.05, ^**^ *p* < 0.01, ^****^ *p* < 0.0001. B), HE and IHC staining of DDAH1, p‐EGFR, and EGFR in subcutaneous xenograft tumors from the S26 cell line expressing the DDAH1 overexpression plasmid (empty vector plasmid is set as control). Scale bar, 100 µm. C), Kaplan–Meier analysis of 3‐year progression‐free survival (PFS), overall survival (OS), recurrence‐free survival (RFS) and distant metastasis‐free survival (DMFS) in 190 DDAH1‐positive patients with LANPC treated with or without nimotuzumab. D), Kaplan–Meier analysis of 3‐year PFS, OS, RFS, and DMFS in 149 DDAH1‐negative patients with LANPC treated with or without nimotuzumab. E), The proposed mechanism model for DDAH1 function in NPC cisplatin resistance. DDAH1 binds to the intracellular domain of EGFR and promotes EGFR dimerization and phosphorylation, thus increases activation of the JAK2‐STAT3 pathway to enhance cisplatin resistance.

To assess whether DDAH1 contributes to cisplatin resistance in NPC cells in vivo, stable DDAH1‐overexpressing S26 cells or control S26 cells were injected into the right axilla of M‐NSG mice to generate an NPC model (Figure **8**A). Tumor size increased in mice injected with DDAH1‐overexpressing cells compared to the control group (Figure 8A). Furthermore, DDAH1 overexpression conferred resistance to cisplatin, as the weights and volumes of the tumors did not decrease significantly following intraperitoneal cisplatin administration (Figure 8A). IHC analysis revealed increased p‐EGFR levels in tumors from mice with DDAH1 overexpression (Figure 8B). Mice treated with both nimotuzumab (tail vein injection) and cisplatin (intraperitoneal injection) showed a significant reduction in tumor weight and volume, even in the presence of DDAH1 overexpression (Figure 8A). These findings indicate that DDAH1 enhances cisplatin resistance, an effect that can be alleviated by nimotuzumab in vivo.

### 2.10 Nimotuzumab may Improve Survival in Patients with LANPC Expressing DDAH1

To further evaluate the potential clinical impact of DDAH1 in regulating NPC cell sensitivity to cisplatin‐based chemotherapy and the potential therapeutic benefits of nimotuzumab, we examined the prognostic value of DDAH1 in patients with LANPC receiving cisplatin‐based chemotherapy. Survival analysis revealed that nimotuzumab could improve the 3‐year PFS and RFS in patients with LANPC and DDAH1‐positive (Figure 8C). However, nimotuzumab did not improve the 3‐year survival outcomes for patients with LANPC and DDAH1‐negative (Figure 8D).

## 3 Discussion

Cisplatin‐based IC followed by CCRT is the recommended treatment for patients with LANPC, which can reduce the risk of disease progression of LANPC.^[^ ^5^, ^6^, ^32^ ^]^ However, ≈20% of patients with NPC develop disease progression due to treatment failure.^[^ ^1^, ^6^, ^10^ ^]^ Drug resistance is a major factor preventing complete remission and contributing to poor survival outcomes for patients with LANPC, creating a significant burden on patients and their families.^[^ ^6^, ^8^ ^]^ In this study, we identified that DDAH1, a novel molecular marker, promoted NPC cisplatin resistance. Most importantly, nimotuzumab could weaken the cisplatin resistance of NPC enhanced by DDAH1, providing a therapeutic target to improve the control of chemotherapy of NPC.

DDAH1 primarily metabolizes ≈80% of endogenous ADMA.^[^ ^33^ ^]^ Notably, DDAH1 overexpression has been observed in many human tumors and is associated with poor survival outcomes.^[^ ^12^, ^13^, ^14^ ^]^ In our study, we found increased DDAH1 expression in tissue samples from IC‐resistant patients with LANPC. Additionally, we observed that overexpression of DDAH1 enhanced cisplatin resistance both in vitro and in vivo. However, we found that DDAH1 is not specifically highly expressed in NPC by comparing with normal nasopharyngeal tissues, which indicates that DDAH1 itself is an enzyme with basic enzyme activity and may not be necessarily important for leading to the occurrence of NPC. Whereas in this study, DDAH1 plays a role in cisplatin resistance of NPC. Cell stemness is one of the important factors that cause chemoresistance,^[^ ^9^ ^]^ we found that DDAH1 increased stemness of NPC cells by increasing *Sox2*, *Oct4* and *Nanog*. The expressions of *Oct4* and *Nanog* of “Vector” NPC cells showed differences in Figures 3D and 7B because the cell lines used in Figures 3D and 7B are at different passage numbers. Even cells derived from the same parental generation will gradually develop cellular heterogeneity during the passage process, there might be differences in gene expression among different individual cells, and such differences may become more pronounced after multiple passages, leading to changes in the protein expression of the overall cell population.^[^ ^34^ ^]^ To investigate the cisplatin resistance mechanism in NPC cells, we focused on the relationship between DDAH1 and EGFR. Our findings revealed that DDAH1 interacts with EGFR in vitro, whose activation is associated with poor survival in many tumors and is highly expressed in most NPC tissues.^[^^]^ Furthermore, we found that overexpression of DDAH1 enhanced cisplatin resistance and made an increase in p‐EGFR by comparing with control group in Figure 7B in vivo, which consolidates the conclusions in vitro. The results in vitro and in vivo above provide a novel perspective on understanding the underlying mechanisms of EGFR‐related diseases. It helps researchers to further explore the disease occurrence and development process from the perspective of DDAH1 and EGFR phosphorylation. Besides, our results showed that DDAH1 enhances the EGFR‐JAK2‐STAT3 signaling pathway, leading to increased cisplatin chemoresistance in NPC cells (Figure 8E). Besides, the typical function of DDAH1 is to regulate intracellular NO concentration by modulating ADMA levels.^[^ ^11^ ^]^ Furthermore, NO concentrations are linked to poor survival and the activation of tumor‐related pathways in various cancers, including breast, colon, and gastric cancers.^[^^]^ However, our findings showed that altering intracellular NO concentration, either by increasing or decreasing it, did not significantly affect the activation of the EGFR‐JAK2‐STAT3 pathway in NPC cells. This suggests that DDAH1 may not enhance the EGFR‐JAK2‐STAT3 pathway through its conventional role of regulating intracellular NO concentration, offering a novel perspective on the activity of DDAH1.

IF co‐localization revealed that DDAH1 was predominantly located intracellularly and on the membrane. When it comes to the interaction between DDAH1 and EGFR, we focused on determining the specific site of interaction of DDAH1 with EGFR. EGFR possesses an extracellular ligand‐binding domain, a single‐pass TM helical domain, and a tyrosine kinase subdomain.^[^^]^ To further investigate, we constructed truncated plasmids for the extracellular domain of EGFR (1‐712) and the intracellular domain of EGFR (646‐1210), as described in previous studies.^[^^]^ Co‐IP results demonstrated that DDAH1 interacts with the intracellular domain of EGFR, which primarily contains tyrosine kinases. Phosphorylation of tyrosine kinases is crucial for the activation of downstream signaling pathways, including JAK2‐STAT3, which play significant roles in tumor growth and progression.^[^ ^16^, ^17^, ^35^ ^]^ Furthermore, we discovered that the interaction between DDAH1 and EGFR promotes EGFR dimerization. Increased EGFR dimer formation can hinder treatment efficacy and contribute to cisplatin resistance. Wild‐type EGFR is activated through binding to extracellular ligands.^[^ ^23^ ^]^ To investigate whether DDAH1 enhances EGFR dimerization and activates the EGFR‐JAK2‐STAT3 pathway through extracellular ligands, we treated DDAH1‐overexpressing NPC cells with nimotuzumab to block the binding of extracellular ligands to EGFR. Blocking this interaction led to a decrease in the activation of the EGFR‐JAK2‐STAT3 signaling pathway and EGFR dimerization, which in turn weakened cisplatin resistance induced by DDAH1.

Our results showed that increased DDAH1 expression in tissue samples from patients with LANPC, IC‐resistant and DDAH1 positivity was associated with poor 3‐year PFS, DMFS, and RFS. These findings may assist clinicians in selecting patients with LANPC at high risk of disease progression due to cisplatin chemoresistance and guide decisions to offer combination therapy and more intensive treatment. Previous studies have also shown that nimotuzumab can improve survival in patients^[^^]^ and may help bypass cisplatin resistance by binding to the extracellular domain of EGFR, thereby blocking ligand binding to EGFR and receptor autophosphorylation when combined with cisplatin derivatives.^[^^]^ To overcome cisplatin resistance, a combination therapy with nimotuzumab may be an effective strategy. However, the indications for such combination therapy remain unclear. For instance, 20% of patients with LANPC experience disease progression within five years, even when treated with nimotuzumab.^[^^]^ Additionally, nimotuzumab is prohibitively expensive for many patients.^[^ ^52^ ^]^ Therefore, it is crucial to identify ideal patients with LANPC and determine clear indications for nimotuzumab use, which would help improve survival rates and reduce the economic burden on patients. Our results demonstrated that nimotuzumab improved the 3‐year PFS and 3‐year RFS of patients with LANPC who were DDAH1‐positive. These findings further support the hypothesis that combination therapy may be essential for patients prone to chemotherapy resistance. Therefore, these studies provide valuable insights into how we can optimize the use of combination therapies, such as nimotuzumab, to enhance efficacy for patients with LANPC susceptible to cisplatin resistance. Our study also emphasizes the importance of extending preclinical research beyond basic model systems for drug development, as human cancers are more complex and may account for differences in drug efficacy.

In conclusion, our study demonstrated that DDAH1 enhances the activation of the EGFR‐JAK2‐STAT3 signaling pathway and promotes EGFR dimerization. The clinical significance of DDAH1 suggests that it could help clinicians predict the risk of cisplatin chemoresistance and disease progression. The EGFR monoclonal antibody nimotuzumab may represent a novel combination strategy for overcoming cisplatin resistance induced by DDAH1. However, we acknowledge that the clinical value of DDAH1 must be further validated in preclinical and large‐scale multicenter studies before its application in clinical settings.

## 4 Experimental Section

### Patients and Samples

To begin, RNA sequencing (RNA‐seq) data were collected from pretreatment NPC tissues of six patients with LANPC at the Sun Yat‐sen University Cancer Center (SYSUCC, Guangzhou, China). Among them, three patients were resistant to IC, while the remaining three were sensitive, baseline data for the selected patients are presented in Table S1 (Supporting Information). Subsequently, 339 formalin‐fixed, paraffin‐embedded NPC specimens from SYSUCC collected between 2019 and 2022 were obtained. All patients were diagnosed with LANPC and had not received any antitumor therapy prior to undergoing biopsy. Patients were staged according to the 8th edition of the American Joint Committee on Cancer (AJCC) Staging Manual. All patients underwent cisplatin‐based IC and CCRT. In this cohort, 177 patients received combination therapy with nimotuzumab, while the remaining 162 patients were treated with IC plus CCRT without any additional antitumor therapy. Baseline data for the selected patients are presented in Table S2 (Supporting Information). The Institutional Ethical Review Board of the SYSUCC approved the use of tumor specimens for this study (G2023‐028‐01) in accordance with the Declaration of Helsinki.

### Cell Culture

Human NPC cell lines (HK1, CNE2, S26, and SUNE2) were generously provided by Professor Musheng Zeng from Sun Yat‐sen University Cancer Center (SYSUCC, Guangzhou, China) and cultured in RPMI‐1640 medium (Gibco, Grand Island, NY, USA) supplemented with 10% fetal bovine serum (FBS; Gibco, Grand island, NY, USA). All cell lines were routinely tested for mycoplasma contamination and were used for fewer than 12 passages. In this study, S26 and HK1 cell lines were choosen to overexpressed DDAH1 because wildtype HK1 and S26 cell lines are two of the NPC cell lines that expressed relatively low DDAH1 (Figure S1A, Supporting Information). Besides, CNE2 was choosen with a moderate expression level of DDAH1 and SUNE2 with a high expression level of DDAH1 for knockdown experiments based on the results of preliminary functional studies. Besides, the knockdown effects in these two cell lines are relatively better, and they have maintained a stable knockdown level during the subculture process (Figure S1A, Supporting Information).

### Plasmid Construction

Plasmid construction was done by GeneCopoeia Company in Guangzhou, China. The information of plasmids used in this study are shown in Table S3 (Supporting Information). The detail of shRNA is shown in Figure S1B–D (Supporting Information).

### Flow Cytometry Side Population (SP) Cell Analysis

Digest the cultured cells with trypsin for 3–5 min, when the cells become round and start to detach, add 1640 medium containing 10% FBS to stop the digestion. Then, the cells were washed twice with pre‐cooled PBS buffer. Centrifuge at 1,000 rpm for 5 min. Finally, resuspend the cells in serum‐free 1640 medium. Keep the samples away from light from this step onward: Divide each cell suspension sample into two parts. For one part, transfer 1 mL of the cell suspension and add 1 µL of emtricitabine (FTC). After incubating in 37 °C, 5% CO₂ incubator for 15 min, add 0.5 µL of Hoechst 33342 (10 mg mL^−1^) and incubated in 37 °C, 5% CO₂ incubator for 90 min and mix the suspension several times every 10 min. The other part of the cell suspension was only added 0.5 µL of Hoechst 33342 (10 mg mL^−1^) and incubate in 37 °C, 5% CO₂ incubator for 90 min and mix the suspension several times every 10 min. Transfer the cells to a 1.5 mL EP tube. Centrifuge at 2,000 rpm for 5 min at 4 °C, then remove the culture medium. Resuspend the cells again in 400 µL of pre‐cooled PBS. At last, dispense the cell suspension into sterile flow cytometry tubes and analyze the samples by using a flow cytometer.

### Mass Spectrometry Analysis

The protein solution was reduced with 0.05 m TCEP for 1 h at 60 °C. Alkylate with 55 mm MMTS for 45 min at room temperature in darkness. Then the protein was added into 10K Millipore, centrifuged at 12,000 rpm at 4 °C for 20 min, and discarded the filtrate. 100 uL UA (8 m urea, 0.1Mtris HCL^−1^, pH 8.5) was added into Millipore, and centrifuged at 12,000 rpm at 4 °C for 20 min twice, discarded the filtrate. 100uL 0.25 m TEAB was added into Millipore and centrifuged at 12,000 rpm at 4 °C for 20 min three times, discarded the filtrate. Then replace a new collection tube. Trypsin was added at 1:50 trypsin‐to‐protein mass ratio for the first digestion overnight and 1:100 trypsin‐to‐protein mass ratio for a second 4 h‐digestion. Centrifuge at 12,000 rpm at 4 °C for 20 min, collect the filtrate. Add 50 µL 0.5 m TEAB into the Millipore and centrifuge at 12,000 rpm for 10 min, collect the filtrate and vacuum dry.

Peptides were dissolved in 0.1% FA and 2% ACN, directly loaded onto a reversed‐phase analytical column (75 um i.d. x 150 mm, packed with Acclaim PepMap RSLC C18, 2um, 100Å, nanoViper), The gradient was comprised of an increase from 5% to 50% solvent B (0.1% FA in 80% ACN) over 40 min, and climbing to 90% in 5 min, then holding at 90% for the 5 min. All at a constant flow rate of 300 nL min^−1^. The MS analysis was performed on Q Exactive hybrid quadrupole‐Orbitrap mass spectrometer (ThermoFisher Scientific).

The peptides were subjected to NSI source followed by tandem mass spectrometry (MS/MS) in Q Exactive^TM^ (Thermo) coupled online to the UPLC. Intact peptides were detected in the Orbitrap at a resolution of 70000. Peptides were selected for MS/MS using NCE setting as 27; ion fragments were detected in the Orbitrap at a resolution of 17500. A data‐dependent procedure that alternated between one MS scan followed by 20 MS/MS scans was applied for the top 20 precursor ions above a threshold ion count of 1E4 in the MS survey scan with 30.0s dynamic exclusion. The electrospray voltage applied was 2.0 kV. Automatic gain control (AGC) was used to prevent overfilling of the ion trap; 1E5 ions were accumulated for the generation of MS/MS spectra. For MS scans, the m/z scan range was 350 to 1800 m z^−1^. Fixed first mass was set as 100 m z^−1^.

### Detection of Intracellular Nitric Oxide Concentration

The method of intracellular nitric oxide concentration detection was done by following the instructions of the Total Nitric Oxide Assay Kit (Yeasen Biotechnology, Shanghai, China).

### Measurement of DDAH1 Activity

DDAH1 activity was assayed as previously described.^[^^]^ The cell lysate was incubated with 4 mmol L ADMA in 0.1 mol L^−1^ sodium phosphate buffer (pH 6.5). The total reaction volume was 0.5 mL, and the incubation was carried out at 37 °C for 3 h. The reaction was halted by adding an equal volume of 10% trichloroacetic acid. Subsequently, the supernatant was boiled with diacetyl monoxime (0.8% wt vol^−1^ in 5% acetic acid) and antipyrine (0.5% wt vol^−1^ in 50% sulfuric acid). The quantity of L‐citrulline generated was measured using spectrophotometric analysis at a wavelength of 466 nm. For the assay blank, the enzyme preparations were heated in a boiling water bath prior to the activity determinations.

### RNA‐Seq and Bioinformatic Analysis

RNA‐Seq data were collected from three patients with LANPC resistant to IC, three patients with LANPC sensitive to IC, and S26 and HK1 NPC cell lines transfected with a DDAH1 overexpression plasmid. RNA‐seq was conducted by Novogene (Tianjin, China). RNA‐Seq reads from paired‐end sequence files (FASTQ files) were mapped to the human reference genome (GRCh38) using Tophat2.^[^ ^54^ ^]^ Gene abundance was determined by calculating count values. The number of fragments within each gene was determined using StringTie software (RRID: SCR_016323) and normalized using the TMM algorithm. Differential expression analysis was conducted using the R EdgeR package (RRID: SCR_012802). Differentially expressed genes with a fold change > 1.2 and a P value < 0.05 were considered significant. Kyoto Encyclopedia of Genes and Genomes (KEGG) pathway analysis (http://www.genome.ad.jp/kegg; RRID: SCR_012773) was performed using the enriched R package (R‐3.4.3). Gene Set Enrichment Analysis (GSEA; http://www.gsea‐msigdb.org/gsea; RRID: SCR_003199) was performed using GSEA software.

### Quantitative Reverse Transcription PCR

RNA and cDNA extraction were performed according to a previous study.^[^ ^55^ ^]^ Reverse transcription was conducted using the GoScript Reverse Transcription System (Promega, Madison, USA). Real‐time PCR was performed using the SYBR Green qPCR reagent (Invitrogen, California, USA) on a LightCycler 480 Instrument II (384‐well, Roche; RRID: SCR_020502) or a CFX96 Real‐Time PCR Detection System (RRID: SCR_018064). GAPDH was used as an internal control for cell line normalization, and the primer sequences used are shown in Table S4 (Supporting Information).

### Western Blot Analysis

Total protein was extracted from the cells using radioimmunoprecipitation assay buffer (Merck Millipore, Billerica, USA) supplemented with protease inhibitors (Roche, Basel, Switzerland) and phosphatase inhibitors (Roche, Basel, Swizerland). The proteins were separated on polyacrylamide gels and transblotted onto polyvinylidene fluoride membranes (Merck Millipore, Massachusetts, USA). Membranes were blocked with 5% skim milk for 1 h and incubated with primary antibodies at 4 °C overnight. Secondary antibodies were added after incubation, and protein bands were detected using a Bio‐Rad ChemiDoc Touch Image System (Bio‐Rad, California, USA; RRID: SCR_021693). The antibodies used are listed in Table S5 (Supporting Information).

### Cross‐Linking Experiment

HK1 and S26 NPC cells were used to analyze EGFR dimerization. The cells were washed three times with ice‐cold phosphate‐buffered saline (PBS) and incubated on ice for 30 min with 3 mm of the cross‐linking reagent bis BS^3^ [bis(sulfosuccinimidyl) suberate]. The reaction was stopped by adding 20 mm Tris (final concentration) to the PBS for 15 min at room temperature (23‐27 °C). Western blotting was used to visualize dimers (> 300 KDa bands) and monomers.

### Immunofluorescence Assay

NPC cells (at a density of 1 × 10⁴) were seeded in 15 mm glass bottom cell culture dishes. Adherent cells were fixed using 4% paraformaldehyde and permeabilized with 0.5% Triton X‐100. The cells were blocked with 5% BSA and incubated with primary antibodies at 4 °C overnight. After washing with PBS, the cells were incubated with the corresponding Alexa Fluor‐conjugated secondary antibodies and washed with PBS. Images were acquired using a confocal laser‐scanning microscope (Carl Zeiss, Oberkochen, Germany). The antibodies used are listed in Table S5 (Supporting Information).

### Co‐Immunoprecipitation

S26 and HK1 cells were collected and lysed them in an immunoprecipitation (IP) lysis buffer (Thermo Fisher Scientific, Waltham, USA) containing a protease inhibitor (Roche, Basel, Switzerland). After centrifugation at 12,000 rpm for 15 min at 4 °C, the cell lysates were incubated with 3 µg of antibody at 4 °C overnight. Protein A/G Magnetic Beads (Thermo Fisher Scientific, Waltham, USA) were used for each reaction, and the mixtures were incubated at room temperature for 1 h. Beads were washed five times with IP lysis buffer and subjected to western blot analysis. The antibodies used are listed in Table S5 (Supporting Information).

### Cell Counting kit 8 Analysis

A commercial Cell Counting Kit 8 (CCK8; Beyotime, Jiangsu, China) was used to analyze cisplatin resistance in NPC cells. Transfected NPC cells, cultured in 96‐well plates, 5000 cells each well. Cells were treated with cisplatin at doses of 0, 4, 8, 16, and 32 µm for 36 h. Afterward, NPC cells were incubated with 10 µL of CCK8 reagent for 1 h. Cell viability was determined by measuring the absorbance at 450 nm using a BioTek Epoch spectrophotometer (Agilent Technologies, California, USA).

### Colony Formation Assays

NPC cells (2000 cells) were seeded in six‐well plates in RPMI 1640 medium (Gibco, Grand Island, NY, USA)with 10% FBS (Gibco, Grand island, NY, USA)and cultured for 10 days. The colonies were fixed with methanol for 30 min and stained with Crystal Violet Staining Solution (Acros Organics, Geel, Belgium)for 20 min. The number of colonies was then counted.

### Apoptotic Cell Death Analysis

The apoptotic rate of NPC cells was analyzed by fluorescein isothiocyanate (FITC) and propidium iodide (PI) staining using a commercial Apoptosis Detection Kit (QCBio Science & Technologies, Shanghai, China). Transfected NPC cells were collected in PBS and suspended in 100 µL of binding buffer. A total of 10 µL of Annexin V‐FITC and PI were simultaneously added to the binding buffer and incubated with NPC cells for 15 min at room temperature in the dark. The cell samples were then loaded onto a FACSCanto II flow cytometer (BD Biosciences, New Jersey, USA), and the cell apoptotic rate was assessed using BD FACSDiva software (BD Biosciences, New Jersey, USA).

### Sphere Formation Assay

NPC cells were seeded onto Corning 96‐well Clear Round Bottom Ultra Low Attachment Microplate (Corning, NY, USA) and cultured in RPMI‐1640 serum‐free medium (Gibco, Grand Island, NY, USA), N21 (Bio‐techne, Minneapolis, MN, USA), EGF (Bio‐techne), and bFGF (Bio‐techne). The cells were incubated for 10 days. The diameters of the spheres were measured using a microscope (OLYMPUS IX73).

### Xenograft Tumor Model

30 NOD‐Prkdcs cidIl2rgem1/Smoc (M‐NSG) immunodeficient mice (female, 15–20 g, 5–6 weeks old) were purchased from the Shanghai Model Organisms Center. Mice are housed in room tempreture (20‐26 °C) and specific pathogen‐free (SPF) environment, their healthy condition are checked every day. For the xenograft tumor models treated with cisplatin alone and cisplatin plus nimotuzumab, 1 × 10⁷ stable S26 cells transfected with a DDAH1 overexpression plasmid (with an empty vector as a control) were mixed with Matrigel (BioCoat, Pennsylvania, USA) and subcutaneously injected into the right axilla of M‐NSG mice (*n* = 5 per group). After tumor establishment, the mice were randomly assigned to three groups: group 1. saline (intraperitoneal injection and tail vein injection), group 2. cisplatin (5 mg kg^−1^, every 3 days, for a total of five doses of cisplatin treatment, intraperitoneally) plus saline (tail vein injection), and group 3. cisplatin (5 mg kg^−1^, every 3 days, for a total of five doses of cisplatin treatment, intraperitoneally) plus nimotuzumab (20 mg kg^−1^, daily, tail vein injection). After 21 days post‐tumor injection, the mice were euthanized by isoflurane inhalation, and the tumors were removed and weighed. Additionally, tumor tissues were collected for hematoxylin and eosin staining, immunohistochemical staining for DDAH1 and EGFR, and phosphorylation of EGFR. All animal experiments were performed in accordance with the National Institutes of Health Guide for the Care and Use of Laboratory Animals and were approved by the Institutional Animal Care and Use Ethics Committee of the SYSUCC (L102012022002J).

### Immunohistochemistry Staining

Paraffin‐embedded tissue sections generated from xenograft mice were deparaffinized, rehydrated, antigen‐retrieved, and the endogenous peroxidase was inactivated. After blocking, the sections were incubated overnight at 4 °C with primary antibodies. An HRP‐conjugated secondary antibody was used to label the slices, followed by incubation with diaminobenzidine. Nuclei were counterstained with hematoxylin for 2 min. The staining intensity of proteins were categorized according to a previous study:^[^ ^56^ ^]^ absent staining as 0, weak as 1, moderate as 2, and strong as 3. The percentage of DDAH1‐stained cells was categorized as follows: no staining = 0, 1%–25% of stained cells = 1, 26%–50% of stained cells = 2, 51%–75% of stained cells = 3, and 76%–100% of stained cells = 4. The proportion and intensity were then multiplied to produce a total score (IHC score) for proteins, ranging from 0 to 12. The primary antibodies used are listed in Table S5 (Supporting Information).

### Statistical Analysis

Statistical analyses were performed using SPSS software (version 22.0; IBM, RRID: SCR_002865) and GraphPad Prism (version 10.0, GraphPad Software Inc.). Data are presented as mean ± standard deviation (SD). The unpaired two‐tailed Student's t‐test or chi‐square test was used to analyze differences between the two groups. The Cox proportional hazards model was employed for univariate and multivariate analysis. One‐way ANOVA, followed by Tukey's multiple comparison test, was performed when there were more than two groups. Survival curves were constructed using Kaplan–Meier curves, with differences analyzed using the log‐rank test. Spearman's rank correlation coefficient (Spearman's r) was used for correlation analysis, with a p‐value < 0.05 considered statistically significant.

## Conflict of Interest

The authors declare no conflict of interest.

## Author Contributions

J.‐H.Y., L.Y., and Q.‐Y.C. contributed equally to this work. H.‐Q. M. and S.‐L. L. conceived the study and provided supervision of the study. J.‐H.Y. wrote the manuscript, performed most of the experiments and data analysis. H.‐Q.M., S.‐L.L., J.‐H.Y., and L.Y. provided review of the study and created figures. J.‐H.Y., L.Y., and S.‐L.L. revised the manuscript. Q.‐Y.C. and L.‐Q.T. provided data curation and conceptualization of the study. K.‐Q.L., L.‐J.L., and Y.‐C.L. participated in some experiments. X.‐Y.L. and X.‐S.S provided some methodology for the study. All authors read and approved the final manuscript.

## Supporting information



Supporting Information

## Data Availability

The data that support the findings of this study are available from the corresponding author upon reasonable request.;
