# Supplementary material for: DDAH1 Promotes Cisplatin Chemoresistance in Patients with Locally Advanced Nasopharyngeal Carcinoma via the EGFR‐JAK2‐STAT3 Pathway
Source: Adv Sci (Weinh). 2025 Jun 19;12(30):e03647. doi: 10.1002/advs.202503647 (PMC12376576; doi:10.1002/advs.202503647)
Supplement: Supplementary file 1 — Supporting Information [file ADVS-12-e03647-s001.pdf]

ADVANCED SCIENCE
Open Access

# Supporting Information

for *Adv. Sci.*, DOI 10.1002/advs.202503647

DDAH1 Promotes Cisplatin Chemoresistance in Patients with Locally Advanced Nasopharyngeal Carcinoma via the EGFR-JAK2-STAT3 Pathway

*Jin-Hao Yang, Li Yuan, Qiu-Yan Chen, Kai-Qi Lan, Liang-Ji Li, Yu-Chen Li, Xiao-Yun Li, Xue-Song Sun, Lin-Quan Tang*, Sai-Lan Liu* and Hai-Qiang Mai**

**Supplementary Table S1. Basic information of 6 NPC patients performed RNA-seq analysis.**

| | Age | Sex | Pretreatment EBV DNA (copies/ml) | T stage | N stage | TNM stage | Sensitivity to induction chemotherapy | Distant metastasis | Recurrence | Death |
|---|---|---|---|---|---|---|---|---|---|---|
| Patient 1 | 38 | Male | 889 | 3 | 3 | 4 | sensitive | No | No | No |
| Patient 2 | 49 | Female | 559 | 3 | 1 | 3 | sensitive | No | No | No |
| Patient 3 | 60 | Male | 1021 | 3 | 1 | 3 | sensitive | No | No | No |
| Patient 4 | 47 | Female | 0 | 3 | 1 | 3 | resistance | Lung | No | No |
| Patient 5 | 50 | Female | 1800 | 3 | 2 | 3 | resistance | Liver | regional | No |
| Patient 6 | 45 | Male | 119 | 4 | 2 | 4 | resistance | No | No | No |

**Supplementary Table S2. Clinical characteristics of 339 NPC patients according to DDAH1 positive and DDAH1 negative.**

| **Characteristics** | **No. of patients** | **Expression of DDAH1** | | ***P* value** |
|---|---|---|---|---|
| | | **DDAH1 negative, n (%)** | **DDAH1 positive, n (%)** | |
| **Age (years)** | | | | |
| <40 | 112 | 57 (30) | 55 (37) | 0.2012 |
| ≥40 | 227 | 133 (70) | 94 (63) | |
| **Sex** | | | | |
| Male | 245 | 137 (72) | 108 (72) | >0.9999 |
| Female | 94 | 53 (28) | 41 (28) | |
| **Nimotuzumab treatment** | | | | |
| No | 162 | 96 (51) | 66 (44) | 0.2744 |
| Yes | 177 | 94 (49) | 83 (56) | |
| **EBV DNA (copies/ml)** | | | | |
| <1500 | 253 | 136(72) | 117(79) | 0.1670 |
| ≥1500 | 86 | 54 (28) | 32 (21) | |
| **VCA-IgA** | | | | |
| <1:80 | 117 | 66 (35) | 51 (34) | >0.9999 |
| ≥1:80 | 222 | 124 (65) | 98 (66) | |
| **EA-IgA** | | | | |
| <1:10 | 77 | 45 (24) | 32 (21) | 0.6957 |
| ≥1:10 | 262 | 145 (76) | 117 (79) | |
| **T Stage** | | | | |
| T1-T3 | 240 | 135 (71) | 105 (70) | 0.9049 |
| T4 | 99 | 55 (29) | 44 (30) | |

| | | | | |
|---|---|---|---|---|
| **N Stage** | | | | |
| N0-N1 | 167 | 93 (49) | 74 (50) | 0.9132 |
| N2-N3 | 172 | 97 (51) | 75 (50) | |
| **TNM Stage** | | | | |
| III | 164 | 84 (44) | 80 (54) | 0.1004 |
| IVA | 175 | 106 (56) | 69 (46) | |
| **Distant metastasis** | | | | |
| No | 319 | 185 (97) | 134 (90) | **0.0048** |
| Yes | 20 | 5 (3) | 15 (10) | |
| **Recurrence** | | | | |
| No | 322 | 186 (98) | 136 (91) | **0.01** |
| Yes | 17 | 4 (2) | 13 (9) | |
| **Death** | | | | |
| No | 326 | 185 (97) | 141 (95) | 0.2561 |
| Yes | 13 | 5 (3) | 8 (5) | |

Abbreviations: DDAH1, dimethylarginine dimethylaminohydrolase; EBV, Epstein-Barr virus; VCA-IgA, viral capsid antigen immunoglobulin A; EA-IgA, early antigen immunoglobulin A; *P* value was determined by chi-square test.

**Supplementary Table S3. Details of plasmid used in the study.**

| Plasmid name | Plasmid description | Plasmid type | Plasmid specification | Plasmid titer | Quantity used of plasmid |
|---|---|---|---|---|---|
| EX-U0918-Lv105 | pLV-pUC Ori-Puro-CMV-DDAH1 (NM_012137) | Overexpression lentiviral vector | 130 μl | 798 ng/μl | 3 |
| EX-NEG-Lv105 | pLV-pUC Ori-Puro-CMV | Control lentivirus vector | 140 μl | 769 ng/μl | 3 |
| EX-U0918-Lv181 | pLV-pUC Ori-Puro-CMV-DDAH1 (NM_012137)-3Flag | Overexpression lentiviral vector | 110 μl | 1,000 ng/μl | 1 |
| EX-NEG-Lv181 | pLV-pUC Ori-Puro-CMV-3Flag | Control lentivirus vector | 125 μl | 980 ng/μl | 1 |
| EX-A8661-Lv120 | pLV-pUC Ori-Puro- CMV- | Overexpression | 150 | 879 | 2 |

| | | | | | |
|---|---|---|---|---|---|
| | EGFR(NM_005228)-3HA | lentiviral vector | μl | ng/μl | |
| CS-A8661-Lv120-05 （EGFR truncated plasmid: 1-712aa） | pLV-pUC Ori-Puro- CMV-EGFR Mut 1 (NM_005228)-3HA | Truncated lentivirus vector | 150 μl | 768 ng/μl | 2 |
| CS-A8661-Lv120-04 （EGFR truncated plasmid: 646-1210aa） | pLV-pUC Ori-Puro- CMV-EGFR Mut 2 (NM_005228)-3HA | Truncated lentivirus vector | 137 μl | 829 ng/μl | 2 |
| EX-A8661-Lv251 | pLV-pUC Ori-Puro- CMV-EGFR(NM_005228)-3Flag | Overexpression lentiviral vector | 100 μl | 997 ng/μl | 1 |
| EX-NEG-Lv120 | pLV-pUC Ori-Puro-CMV-3HA | Control lentivirus vector | 100 μl | 978 ng/μl | 2 |
| EX-NEG-Lv251 | pLV-pUC Ori-Puro-CMV-3Flag | Control lentivirus vector | 120 μl | 980 ng/μl | 1 |
| CSHCTR001-LVRU6P | pLV-pUC Ori-Puro-U6-shNC-SV40 | Control lentivirus vector | 110 μl | 1,000 ng/μl | 2 |
| HSH171800-LVRU6P-a | pLV-pUC Ori-Puro-U6-shDDAH1#1-SV40 | shRNA lentivirus vector | 110 μl | 998 ng/μl | 2 |
| HSH171800-LVRU6P-b | pLV-pUC Ori-Puro-U6-shDDAH1#2-SV40 | shRNA lentivirus vector | 100 μl | 965 ng/μl | 2 |
| EX-U0918-Lv181-01 | pLV-pUC Ori-Puro-CMV-DDAH1$^{C273S}$ (NM_012137)-3Flag | Overexpression lentiviral vector | 100 μl | 900 ng/μl | 1 |

**Supplementary Table S4. The sequences for qRT-PCR primers.**

| Gene name | Forward (5'-3') | Reverse (5'-3') |
|---|---|---|
| DDAH1 | CAAAAGGACAAATCAACGAGGTG | TGTGCAGATTCACTAGACCCAA |
| Sox2 | GCCGAGTGGAAACTTTTGTCG | GGCAGCGTGTACTTATCCTTCT |
| Oct4 | CTGGGTTGATCCTCGGACCT | CCATCGGAGTTGCTCTCCA |
| Nanog | TTTGTGGGCCTGAAGAAAACT | AGGGCTGTCCTGAATAAGCAG |
| GAPDH | CCAGAACATCATCCCTGCCT | CCTGCTTCACCACCTTCTTG |

**Supplementary Table S5. List of antibodies in this study.**

| Antibodies | Company | Catalog no. | Dilution |
|---|---|---|---|
| **Western blotting** | | | |
| Anti DDAH1 Antibody, Rabbit monoclonal | Abcam | ab180599 | 1:10000 |
| EGF Receptor (D38B1) XP, Rabbit mAb | CST | 4267S | 1:1000 |
| Phospho-EGF Receptor (Tyr1068) (D7A5) XP, Rabbit mAb | CST | 3777S | 1:1000 |
| Stat3 (24H6), Mouse mAb | CST | 9139S | 1:1000 |
| Phospho-Stat3 (Tyr705) (D3A7) XP, Rabbit mAb | CST | 9145S | 1:1000 |
| Anti JAK2 Antibody, Rabbit monoclonal | Abcam | ab108596 | 1:5000 |
| Anti JAK2 (phospho Y1007+Y1008) Antibody, Rabbit monoclonal | Abcam | ab32101 | 1:1000 |
| Anti HA-Tag, Mouse mAb | Abclonal | AE008 | 1:2000 |
| Anti-Flag M2 Antibody, Rabbit mAb | CST | 14793S | 1:1000 |
| Alpha Tubulin Antibody, Rabbit Polyclonal | Proteintech | 11224-1-AP | 1:3000 |
| Anti-Rabbit IgG, HRP-linked Antibody | CST | 7074S | 1:2000 |
| Anti-Mouse IgG, HRP-linked Antibody | CST | 7076S | 1:2000 |
| Oct4 Antibody, Rabbit mAb | CST | 2750S | 1:1000 |
| Nanog (D73G4) XP, Rabbit mAb | CST | 4903S | 1:2000 |
| Sox2 Antibody, Rabbit Polyclonal | Proteintech | 11064-1-AP | 1:1000 |
| **Immunofluorescence** | | | |
| Anti DDAH1 Antibody, Rabbit monoclonal | Abcam | ab180599 | 1:100 |
| Anti EGFR Antibody, Mouse monoclonal | Abcam | ab30 | 1μg/ml |
| Anti HA-Tag, Mouse mAb | Abclonal | AE008 | 1:50 |
| Anti-Flag M2 Antibody, Rabbit mAb | CST | 14793S | 1:400 |
| Phospho-Stat3 (Tyr705) (D3A7) XP, Rabbit mAb | CST | 9145S | 1:200 |
| Goat Anti-Mouse, Alexa Fluor 488 IgG | Abcam | ab150113 | 1:500 |
| Goat Anti-Rabbit, Alexa Fluor 594 IgG | Abcam | ab150080 | 1:500 |
| Goat Anti-Rabbit, Alexa Fluor 488 IgG | Abcam | ab150077 | 1:500 |
| **Co-Immunoprecipitation** | | | |
| Anti DDAH1 Antibody, Rabbit monoclonal | Abcam | ab180599 | 1:40 |
| EGF Receptor (D38B1) XP, Rabbit mAb | CST | 4267S | 1:100 |
| **Immunohistochemical staining** | | | |
| Anti DDAH1 Antibody, Rabbit monoclonal | Abcam | ab180599 | 1:50 |
| Phospho-EGF Receptor (Tyr1068) (D7A5) XP, Rabbit mAb | CST | 3777S | 1:500 |
| EGF Receptor (D38B1) XP, Rabbit mAb | CST | 4267S | 1:50 |

**Supplementary Table S6. Univariate and multivariable Cox regression analysis of DDAH1 expression and survival in 339 patients.**

| | Univariate analysis | | | Multivariate analysis | | |
|---|---|---|---|---|---|---|
| **Variable** | ***HR*** | ***95%CI*** | ***P* value** | ***HR*** | ***95%CI*** | ***P* value** |
| **Progression-free survival** | | | | | | |
| DDAH1 expression (IHC score>0 vs. 0) | 3.317 | 1.698-6.478 | **<0.001** | 4.482 | 2.247-8.943 | **<0.001** |
| TMN stage (IVA vs. III) | 3.075 | 1.504-6.288 | **0.002** | | | NS |
| T stage (T4 vs. T1-3) | 1.505 | 0.806-2.809 | 0.199 | | | NS |
| N stage (N2-3 vs. N0-1) | 2.810 | 1.408-5.606 | **0.003** | | | NS |
| Age (≥40 vs. <40 years) | 1.283 | 0.656-2.506 | 0.466 | | | NS |
| Gender (Female vs. male) | 0.710 | 0.340-1.485 | 0.363 | | | NS |
| VCA-IgA (≥1:80 vs. <1:80) | 1.193 | 0.620-2.298 | 0.598 | | | NS |
| EA-IgA (≥1:10 vs. <1:10) | 0.724 | 0.370-1.416 | 0.345 | | | NS |
| EBV DNA (≥1500 vs. <1500) | 3.300 | 1.796-6.061 | **<0.001** | 2.925 | 1.326-6.455 | **0.008** |
| **Recurrence-free survival** | | | | | | |
| DDAH1 expression (IHC score>0 vs. 0) | 4.137 | 1.349-12.69 | **0.013** | 5.728 | 1.751-18.74 | **0.004** |
| TNM stage (IVA vs. III) | 1.818 | 0.671-4.924 | 0.240 | | | NS |
| T stage (T4 vs. T1-3) | 2.178 | 0.839-5.650 | 0.110 | | | NS |
| N stage (N2-3 vs. N0-1) | 0.879 | 0.339-2.283 | 0.792 | | | NS |
| Age (≥40 vs. <40 years) | 0.564 | 0.218-1.462 | 0.239 | | | NS |
| Gender (Female vs. male) | 0.795 | 0.259-2.438 | 0.688 | | | NS |
| VCA-IgA (≥1:80 vs. <1:80) | 0.769 | 0.293-2.021 | 0.595 | | | NS |
| EA-IgA (≥1:10 vs. <1:10) | 0.423 | 0.161-1.112 | 0.081 | | | NS |
| EBV DNA (≥1500 vs. <1500) | 1.652 | 0.610-4.469 | 0.323 | | | NS |
| **Distant metastasis** | | | | | | |

| | | | | | | |
|---|---|---|---|---|---|---|
| **survival** | | | | | | |
| DDAH1 expression (IHC score>0 vs. 0) | 3.899 | 1.417-10.73 | **0.008** | 4.696 | 1.674-13.17 | **0.003** |
| TNM stage (IVA vs. III) | 2.999 | 1.087-8.272 | **0.034** | | | NS |
| T stage (T4 vs. T1-3) | 1.638 | 0.669-4.012 | 0.280 | | | NS |
| N stage (N2-3 vs. N0-1) | 4.106 | 1.371-12.30 | **0.012** | | | NS |
| Age (≥40 vs. <40 years) | 2.041 | 0.682-6.105 | 0.202 | | | NS |
| Gender (Female vs. male) | 0.287 | 0.066-1.235 | 0.094 | | | NS |
| VCA-IgA (≥1:80 vs. <1:80) | 1.610 | 0.585-4.430 | 0.357 | | | NS |
| EA-IgA (≥1:10 vs. <1:10) | 1.207 | 0.404-3.612 | 0.736 | | | NS |
| EBV DNA (≥1500 vs. <1500) | 2.459 | 1.017-5.943 | **0.046** | | | NS |

Abbreviations: *HR*, hazard ratio; NS, not significant. Bold values indicate *P* <0.05, *P* value was determined by Cox regression analysis.

A

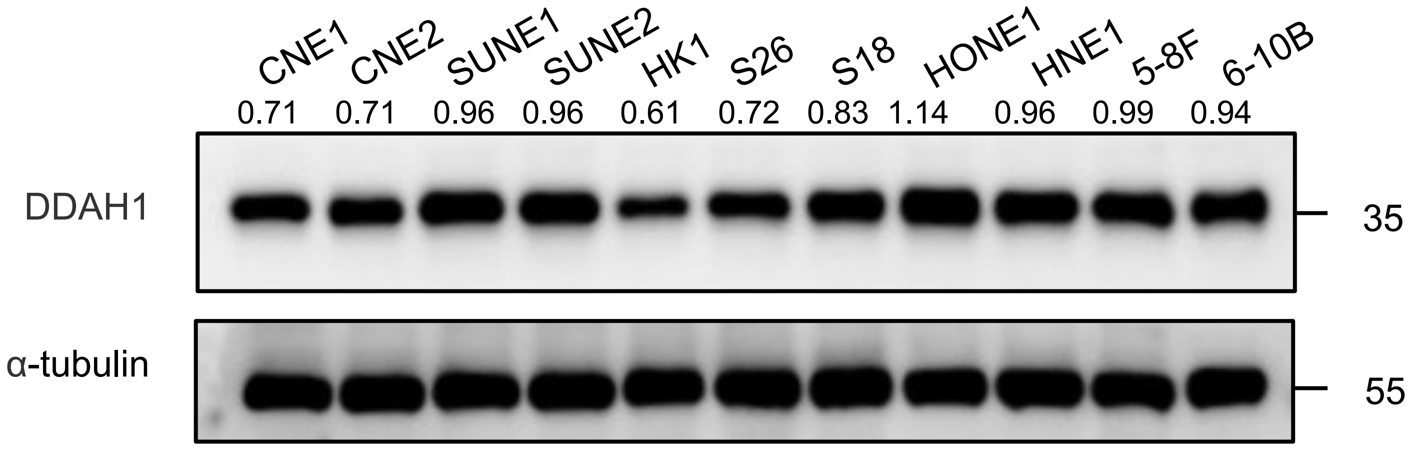


B

**Vector Information for CSHCTR001-LVRU6P**

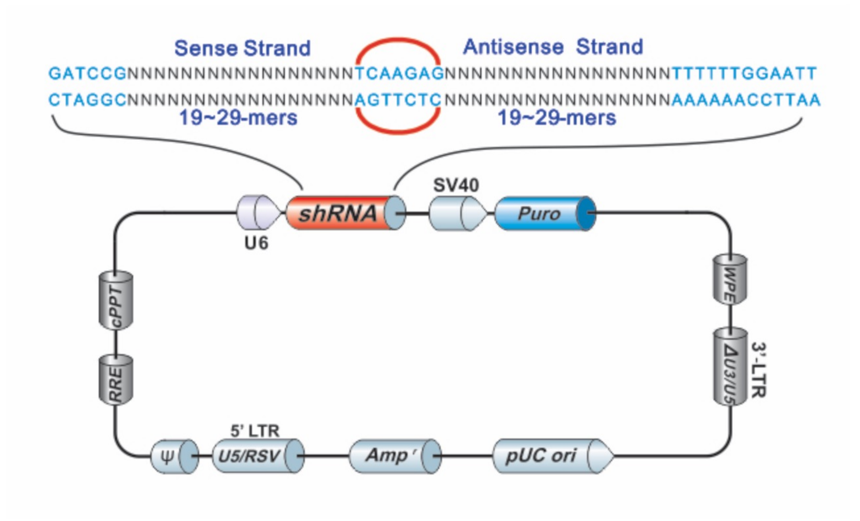


**shRNA Target Sequences**

| Clone Name | Symbol | Location | Length | Target Sequence |
|---|---|---|---|---|
| CSHCTR001-LVRU6P(OSNEG20) | | | 19 | GCTTCGCGCCGTAGTCTTA |

C

**Vector Information for HSH171800-LVRU6P-a**

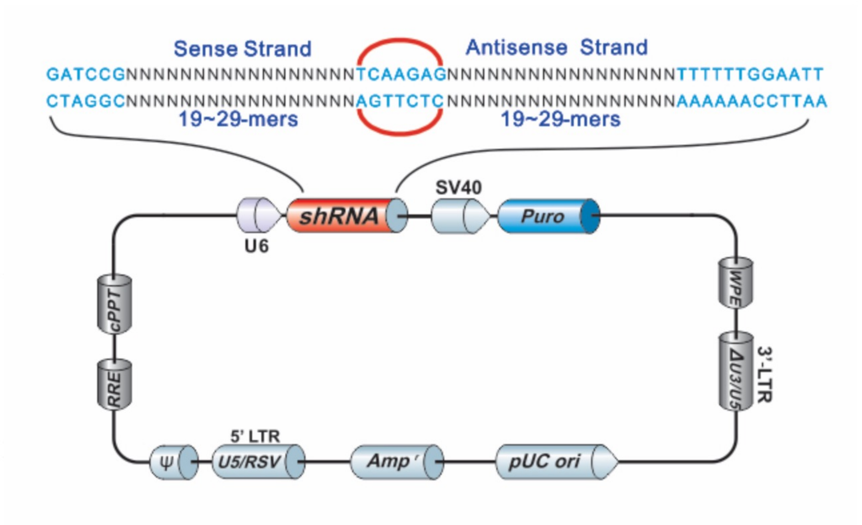


**shRNA Target Sequences**

| Clone Name | Symbol | Location | Length | Target Sequence |
|---|---|---|---|---|
| HSH171800-LVRU6P-a(OS722422) | DDAH1 | 274 | 21 | GCTGAAATCTTGGCTGATACT |

**Vector Information for HSH171800-LVRU6P-b**

D

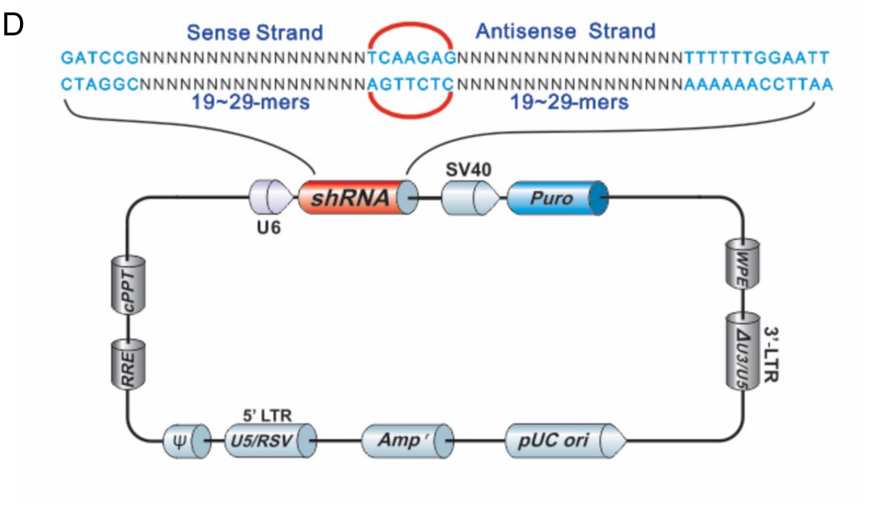


**shRNA Target Sequences**

| Clone Name | Symbol | Location | Length | Target Sequence |
|---|---|---|---|---|
| HSH171800-LVRU6P-b(OS722423) | DDAH1 | 367 | 21 | GGGCCTAACCTGATCGCAATT |

**Supplementary Figure S1. A.** Western blot analysis shows DDAH1 expression in different nasopharyngeal carcinoma (NPC) cell lines. **B,** Vector information for shNC. **C,** Vector information for shDDAH1#1 (NM_001330655.2). **D,** Vector information for shDDAH1#2 (NM_001330655.2).

A

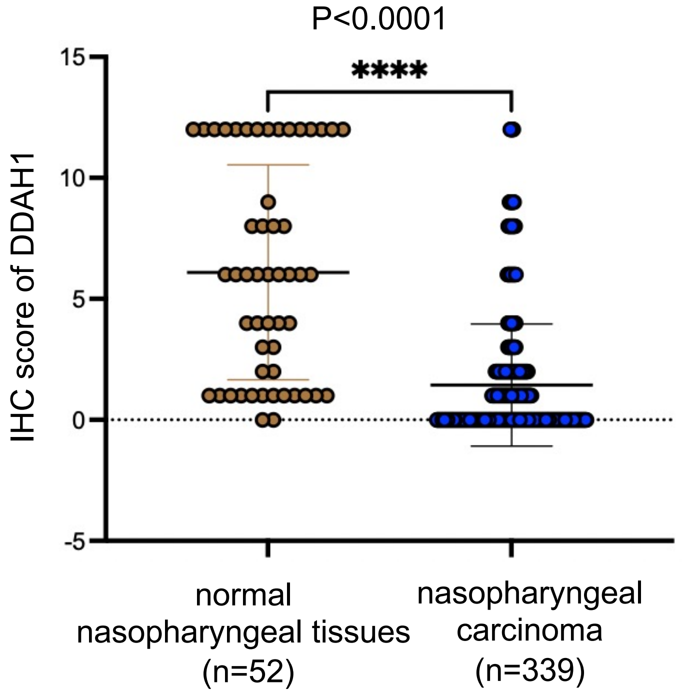


B

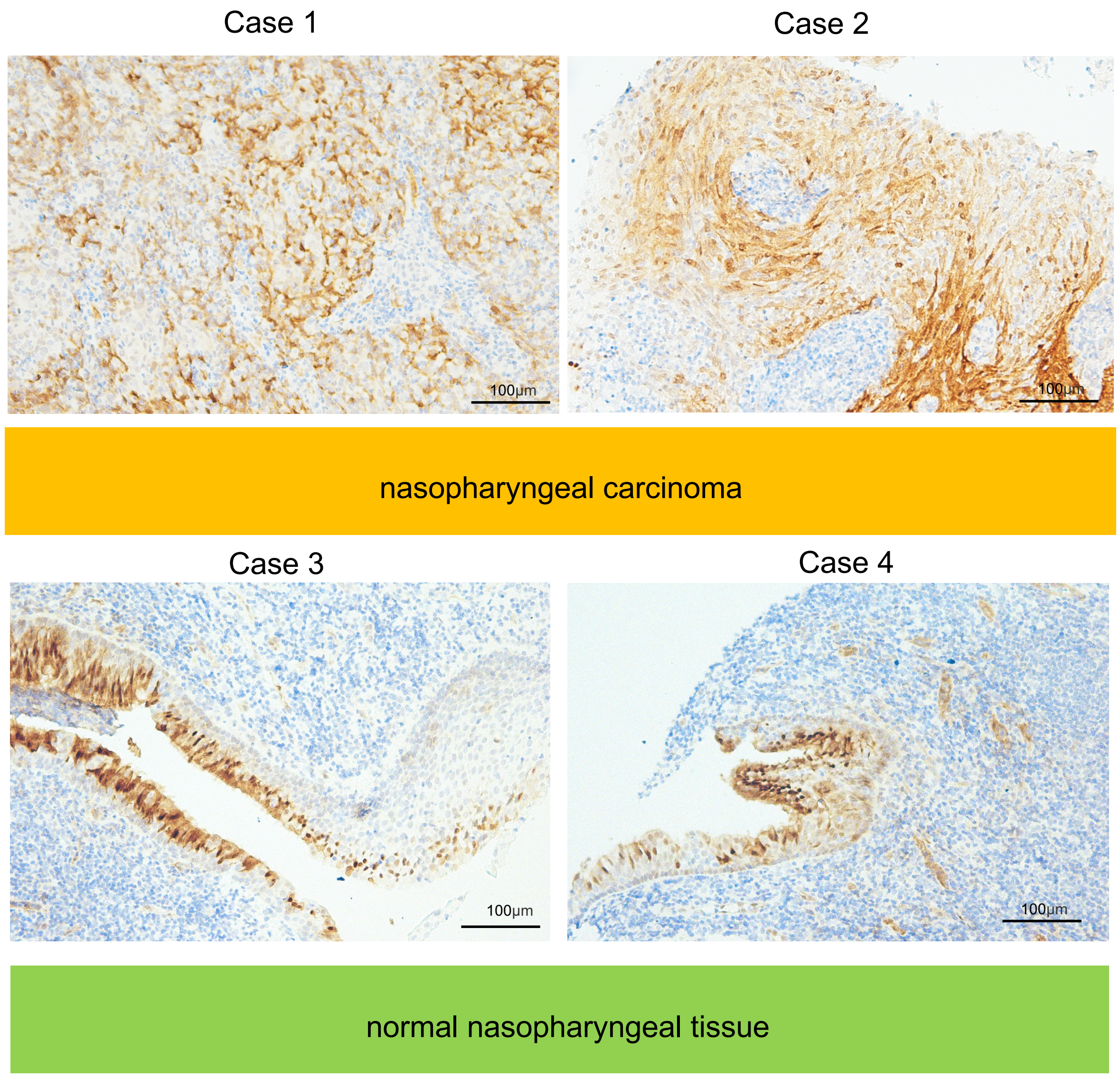


**Supplementary Figure S2. A,** Comparison of Immunohistochemistry (IHC) scores of DDAH1 in 52 normal nasopharyngeal tissues and 339 nasopharyngeal carcinoma (NPC) tissues. Significance was calculated by using unpaired two-tailed Student's t-test. ****P<0.0001. **B,** IHC characteristics of DDAH1 in NPC tissues and normal nasopharyngeal tissues. Scale bar, 100 μm.

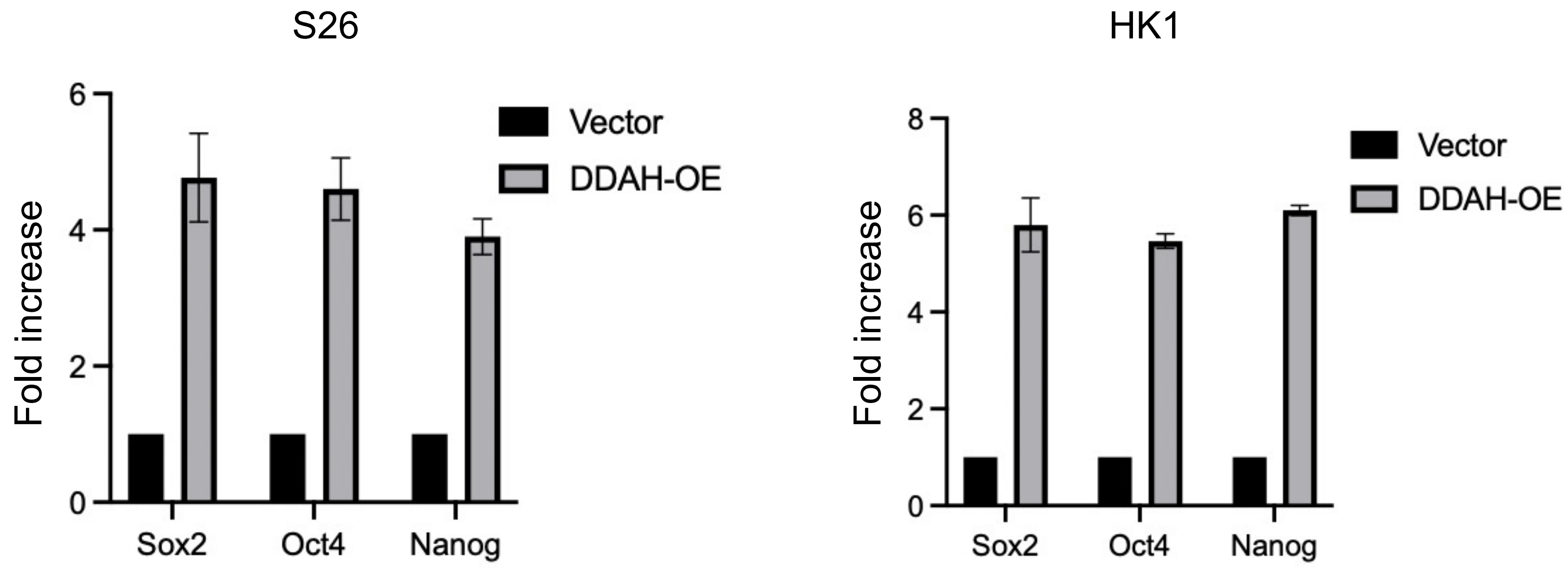


**Supplementary Figure S3** Quantitative real-time PCR (qPCR) showed the relative fold increase of *Sox2, Oct4* and *Nanog* in S26 and HK1 cell lines expressing DDAH1 overexpressing plasmid (empty vector plasmid was set as control).

| Gene name | Mass [kDa] | Score | HK1-OE/HK1-VE | HK1-OE/HK1-VE_DiffStat |
|---|---|---|---|---|
| DDAH1 | 31.121 | 131.67 | 12.406963 | up |
| S100A7 | 11.471 | 12.685 | 4.83483988 | up |
| IGKV4-1 | 13.38 | 13.02 | 4.20559166 | up |
| PRPSAP1 | 39.393 | 9.5893 | 3.746223565 | up |
| IGKV2-29 | 13.085 | 215.24 | 3.63287728 | up |
| HNRNPU | 90.583 | 48.56 | 3.630150717 | up |
| DHX9 | 140.96 | 13.998 | 3.375739645 | up |
| PRPS1 | 34.834 | 76.423 | 3.086253935 | up |
| HNRNPK | 50.976 | 20.795 | 2.846505739 | up |
| RPS18 | 17.718 | 102.85 | 2.820684083 | up |
| EGFR | 134.28 | 22.372 | 2.028589278 | up |
| PRDX1 | 22.11 | 11.944 | 1.973872561 | up |
| ACAP2 | 88.028 | 12.587 | 1.958069727 | up |
| TUBB4B | 49.83 | 66.004 | 1.837987858 | up |
| RPS2 | 31.324 | 32.19 | 1.832478811 | up |
| SLC25A5 | 32.852 | 29.183 | 1.742959023 | up |
| RBM10 | 103.53 | 11.388 | 1.622368978 | up |
| IGKV2-24 | 13.079 | 6.8686 | 1.540326071 | up |
| WDR77 | 36.724 | 124.74 | 1.510881347 | up |
| DDX5 | 69.147 | 26.132 | 0.648799706 | down |
| TRIM21 | 54.169 | 200.89 | 0.583541662 | down |
| RPL7 | 29.225 | 78.884 | 0.569574679 | down |
| BOLA2 | 10.116 | 12.076 | 0.540942483 | down |
| UBA52 | 14.728 | 11.861 | 0.532830039 | down |
| S100A9 | 13.242 | 73.191 | 0.528612681 | down |
| EIF4B | 69.15 | 80.303 | 0.506411363 | down |
| HSPA5 | 72.332 | 17.031 | 0.47922754 | down |
| RPS19 | 16.06 | 18.744 | 0.472149185 | down |

**Supplementary Figure S4** Results of mass spectrometry (MS) detection showed the combination of DDAH1 and EGFR in HK1 cell lines expressing DDAH1 overexpressing plasmid (empty vector plasmid was set as control).

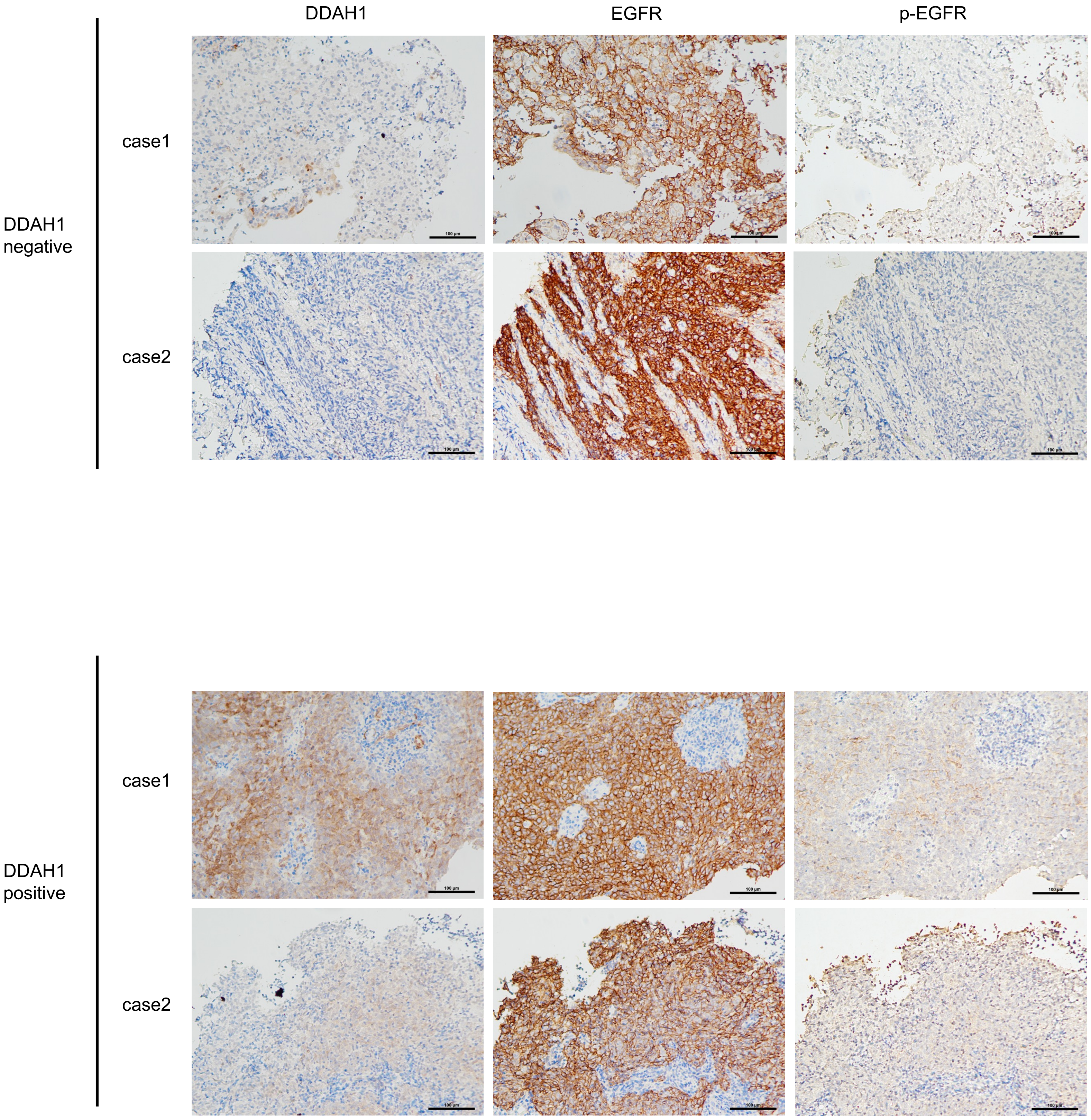


**Supplementary Figure S5** Immunohistochemical (IHC) characteristics of DDAH1, EGFR, p-EGFR (TYR1068) in the patients with locally advanced nasopharyngeal carcinoma (LANPC) with positive and negative DDAH1 expression. Scare bar, 100μm**.**

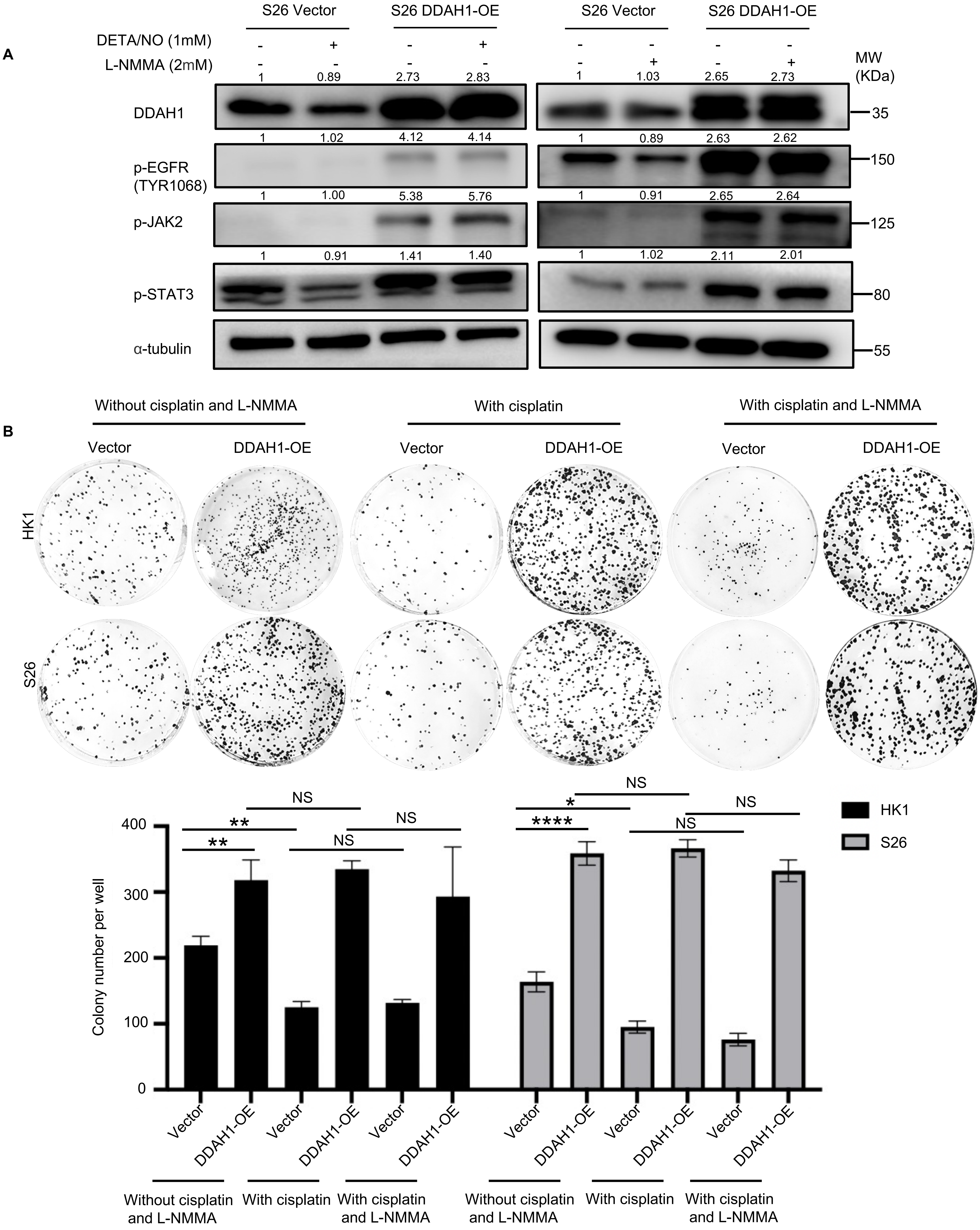


**Supplementary Figure S6 A.** Western blot showed the expression of p-EGFR, p-JAK2 and p-STAT3 in S26 nasopharyngeal carcinoma (NPC) cell lines expressing DDAH1 overexpressing plasmid (and empty vector) treated with DETA/NO for 0 hour and 24 hours and L-NMMA for 0 hour and 1 hour. **B.** Quantified results of colony formation assays in HK1 and S26 cell lines with DDAH1 overexpressing plasmid (and empty vector plasmid was set as control) treated with PBS alone and cisplatin at 5 μM for 36 hours plus PBS for 1 hour and cisplatin at 5 μM for 36 hours plus L-NMMA at 2 mM for 1 hour. The data were presented as mean ± SD and were representative of 3 independent experiments. Significances were calculated by one-way ANOVA with Tukey's multiple comparisons. NS, no significance, *P<0.05, **P<0.01, ****P<0.0001.

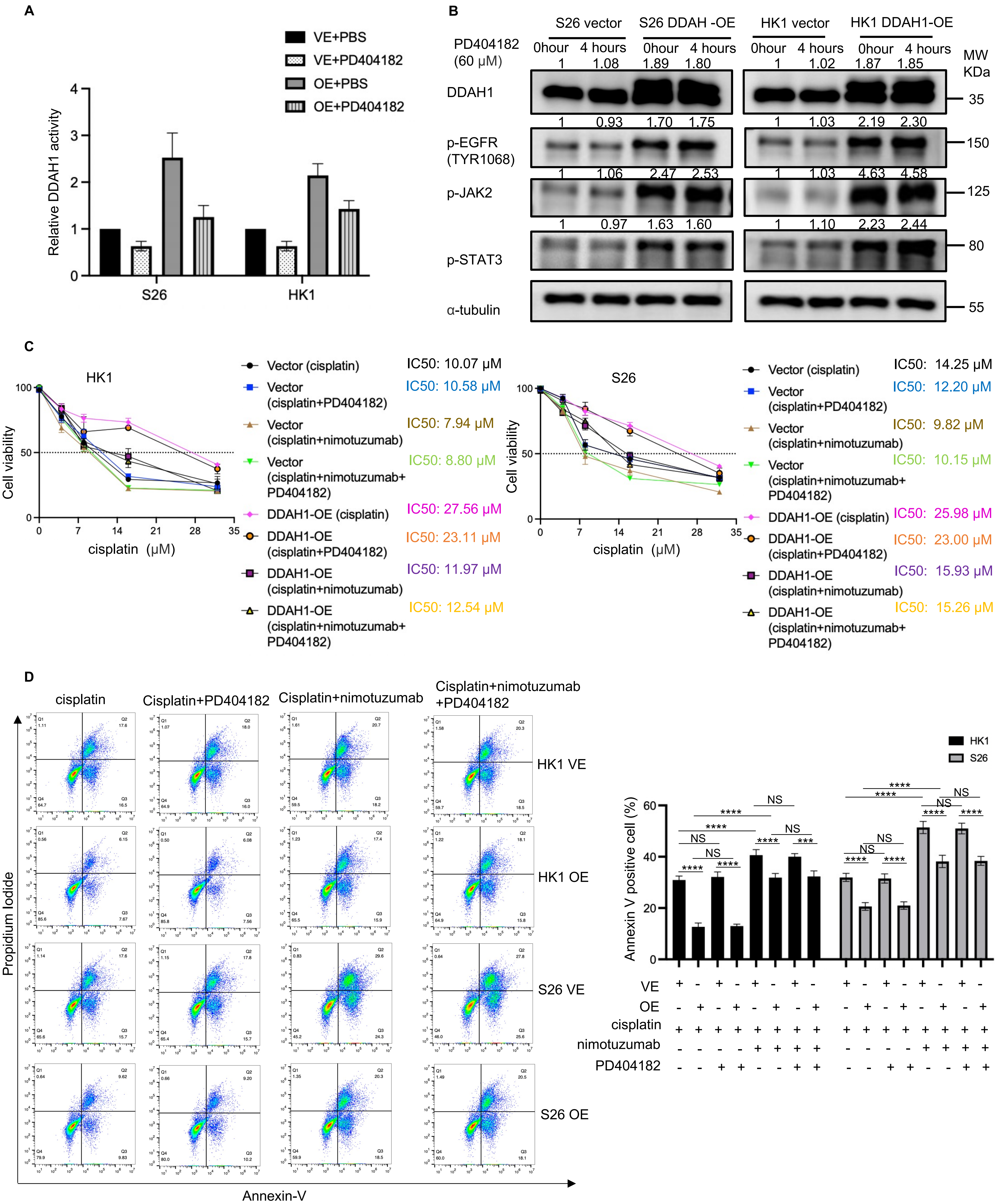


**Supplementary Figure S7. A,** DDAH1 relative activity of DDAH1 overexpression and vector for S26 and HK1 NPC cell lines treated with PD404182 at 60 μM for 4 h and PBS is set for control. "VE" is abbreviation for vector, "OE" is abbreviation for DDAH1-OE. **B,** Western blot analyses show the expression of DDAH1, p-EGFR (TRY1068), p-JAK2, p-STAT3 in S26 and HK1 NPC cell lines with vector, DDAH1 overexpression when they are treated with PD404182 at 60 μM for 4 h. **C,** CCK8 analysis for S26 and HK1 cell lines with DDAH1 overexpression and vector in cisplatin plus PBS group, cisplatin, nimotuzumab plus PBS group, cisplatin, PD404182 plus PBS group and cisplatin, nimotuzumab plus PD404182 group. Cisplatin is given at the indicated concentrations (0, 4, 8, 16, and 32 μM) for 36 h, nimotuzumab is given at 200 μg/mL for 36 h, PD404182 is given at at 60 μM for 4 h**.** The data are presented as mean ± SD and are representative of three independent experiments. **D,** Flow cytometry analysis for S26 and HK1 cell lines with DDAH1 overexpression and vector in cisplatin plus PBS group, cisplatin plus nimotuzumab group, cisplatin plus PD404182 group and cisplatin, nimotuzumab plus PD404182 group. Cisplatin is given at the indicated concentrations 8 μM for 36 h, nimotuzumab is given at 200 μg/mL for 36 h, PD404182 is given at at 60 μM for 4 h. The data are presented as mean ± SD and are representative of three independent experiments. Significances are calculated by one-way ANOVA with Tukey's multiple comparisons. NS, no significance, ***P<0.001, ****P<0.0001. "VE" is abbreviation for vector, "OE" is abbreviation for DDAH1-OE.

Supplementary Figure S8 for unprocessed blots

**Figure 1H**

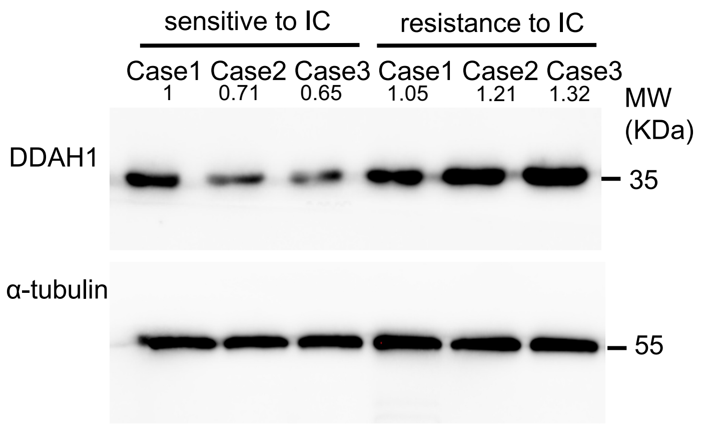


**Figure 2A**

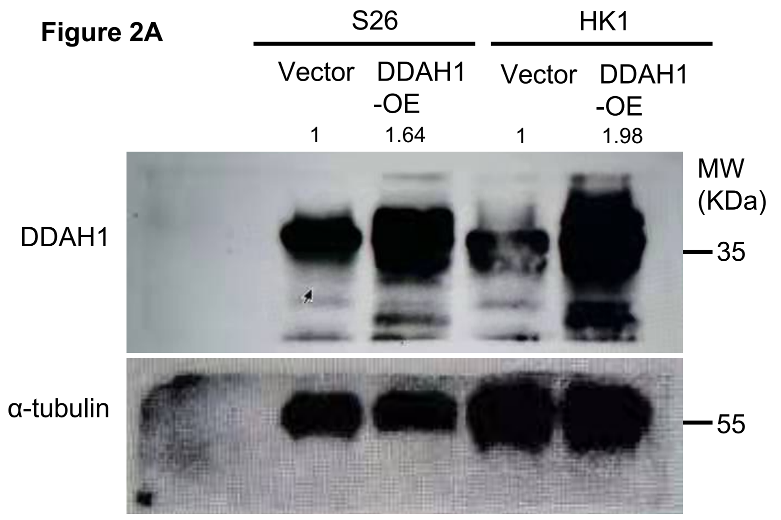


**Figure 2E**

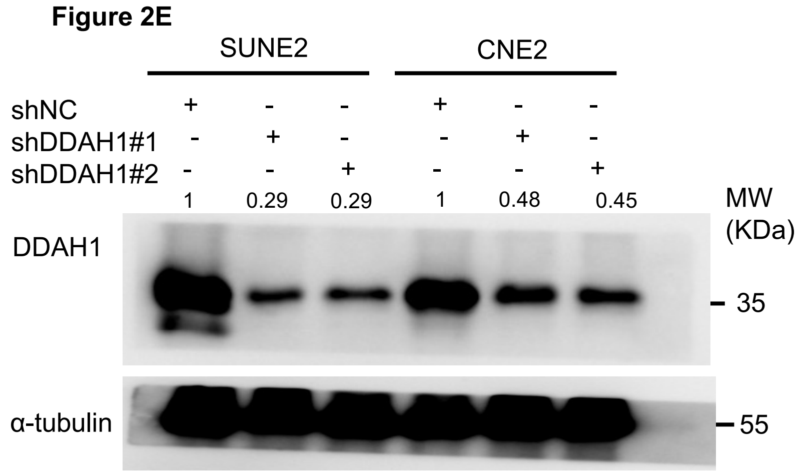


**Figure 3D**

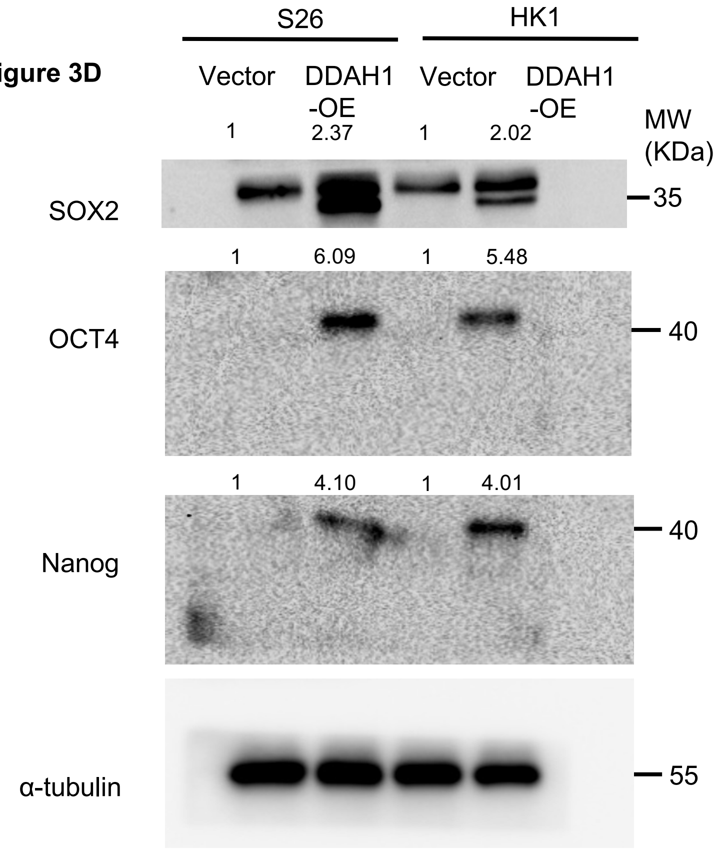


**Figure 3F**

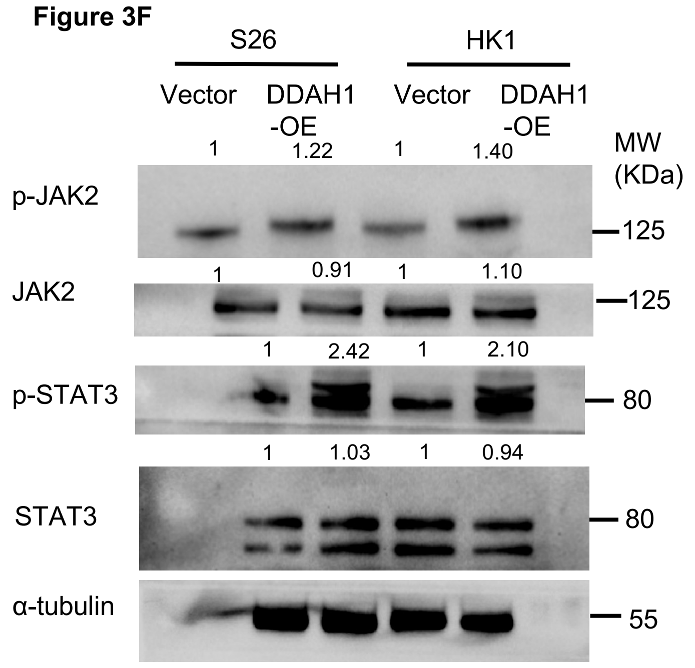


**Figure 3I**

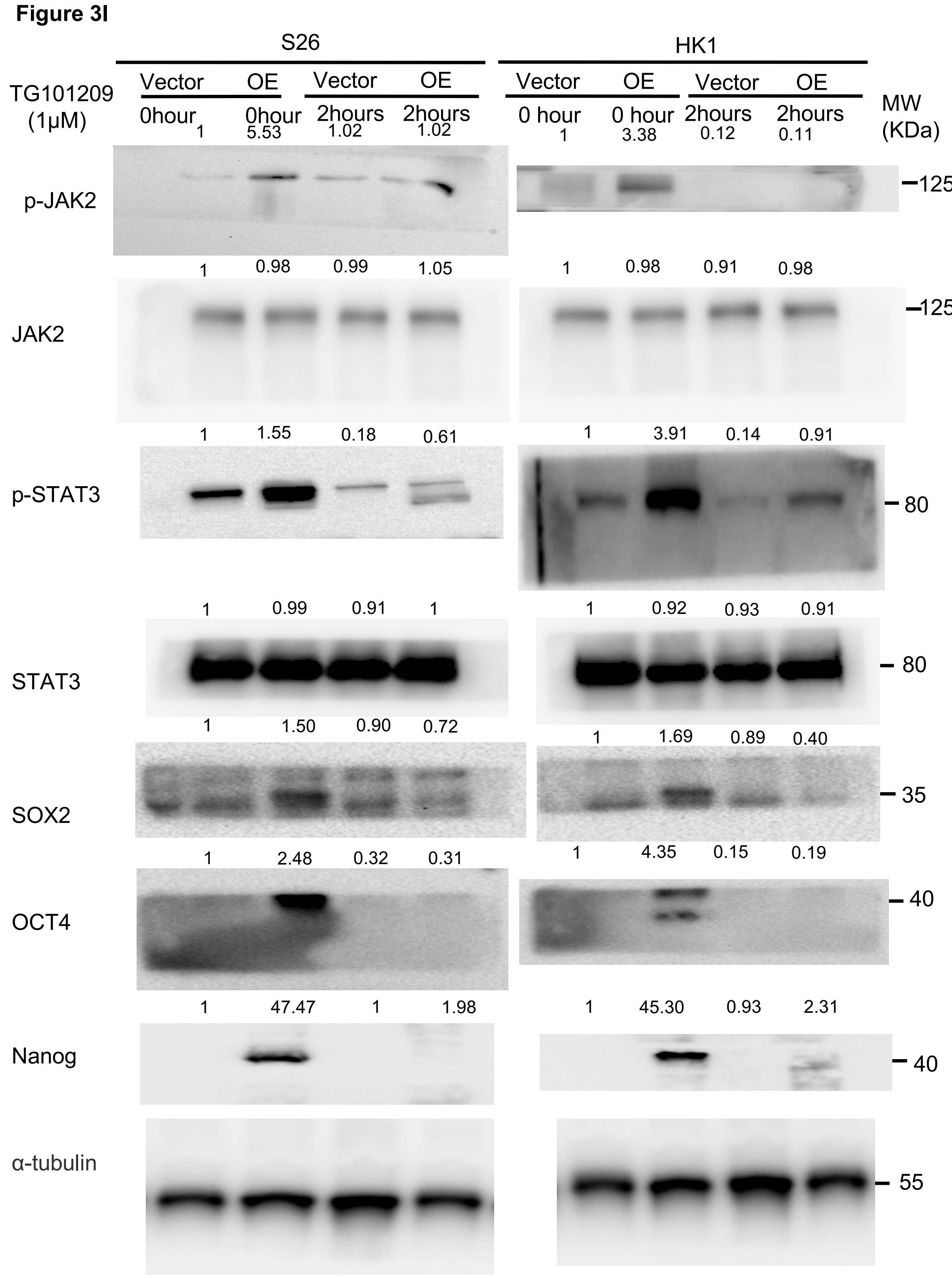


**Supplementary Figure S8.** Unprocessed blots for Figure 1H, Figure 2A, Figure 2E, Figure 3D, Figure 3F and Figure 3I in the manuscript.

Supplementary Figure S9 for unprocessed blots

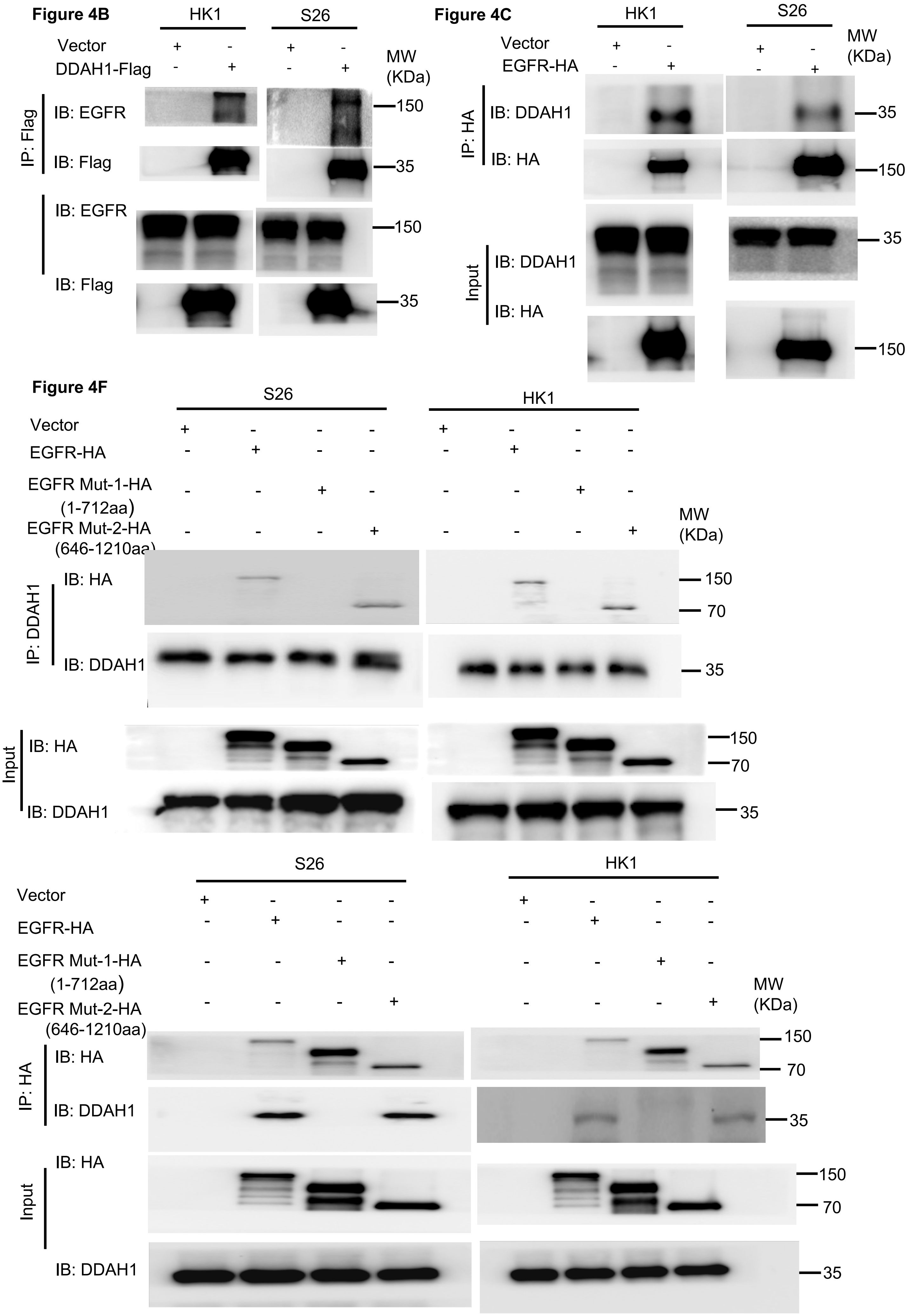


**Supplementary Figure S9.** Unprocessed blots for Figure 4B, Figure 4C and Figure 4F in the manuscript.

Supplementary Figure S10 for unprocessed blots

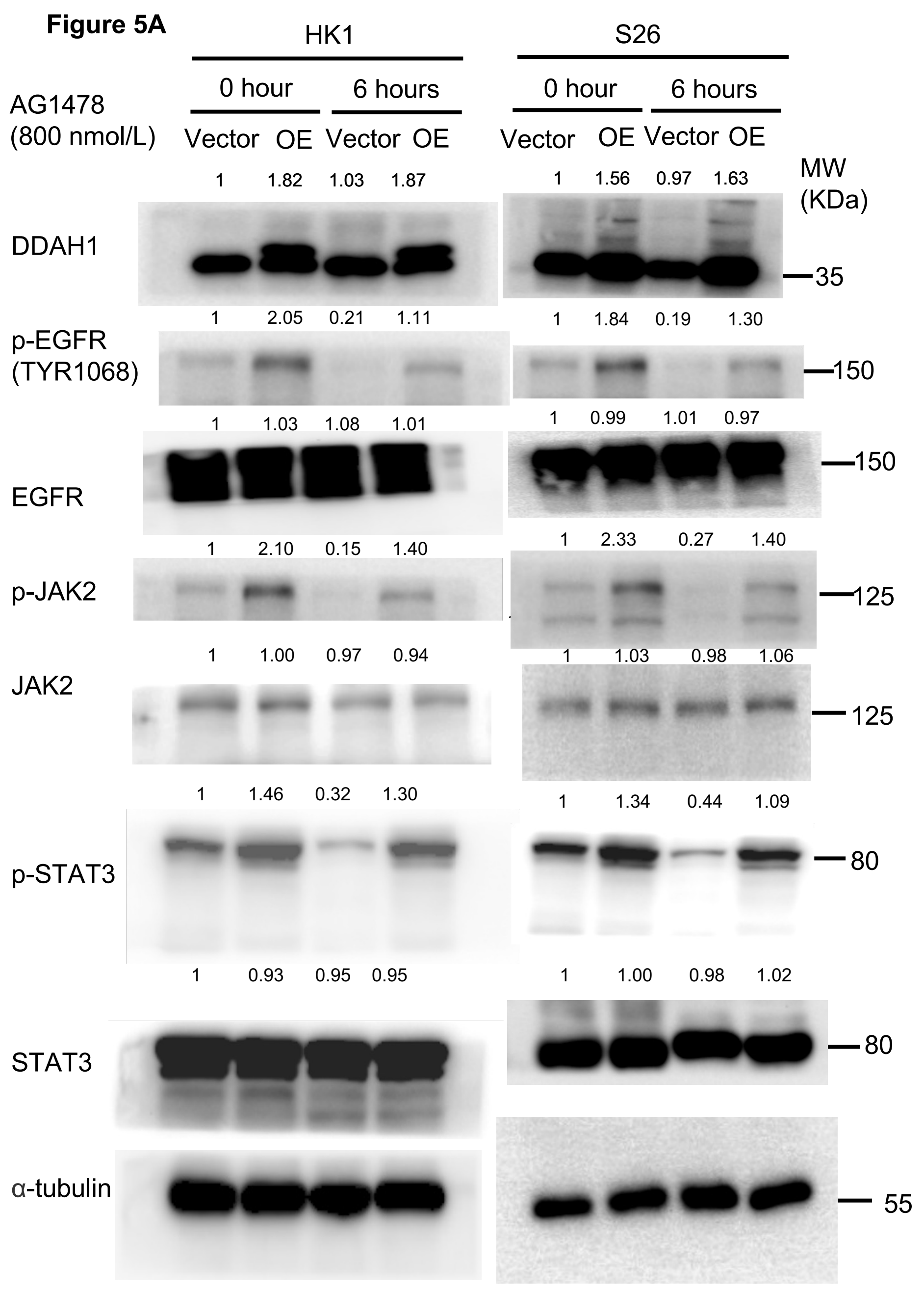


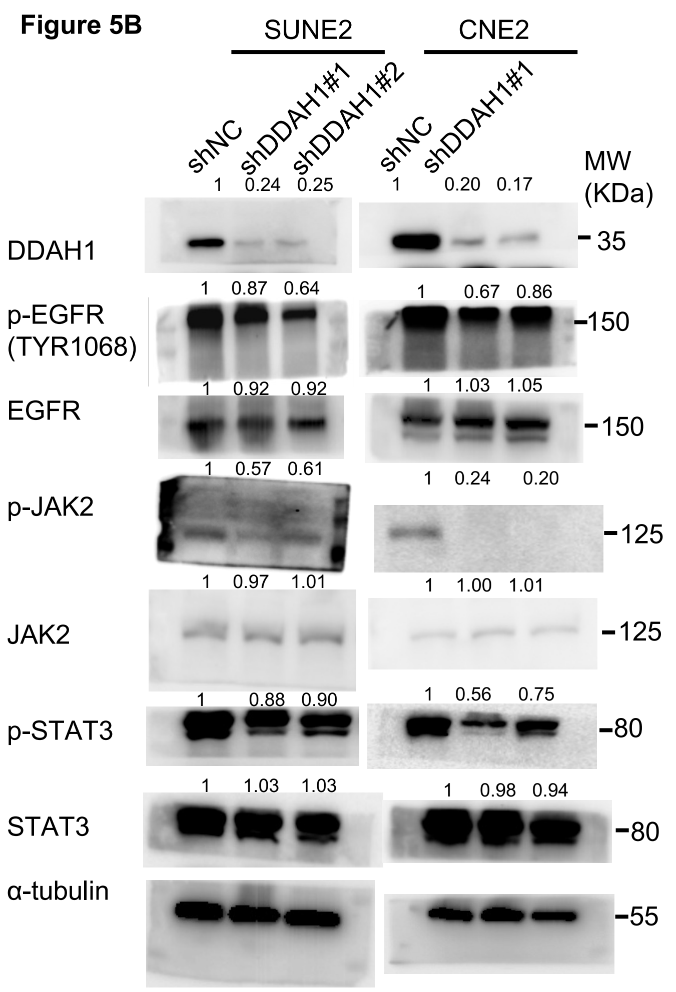


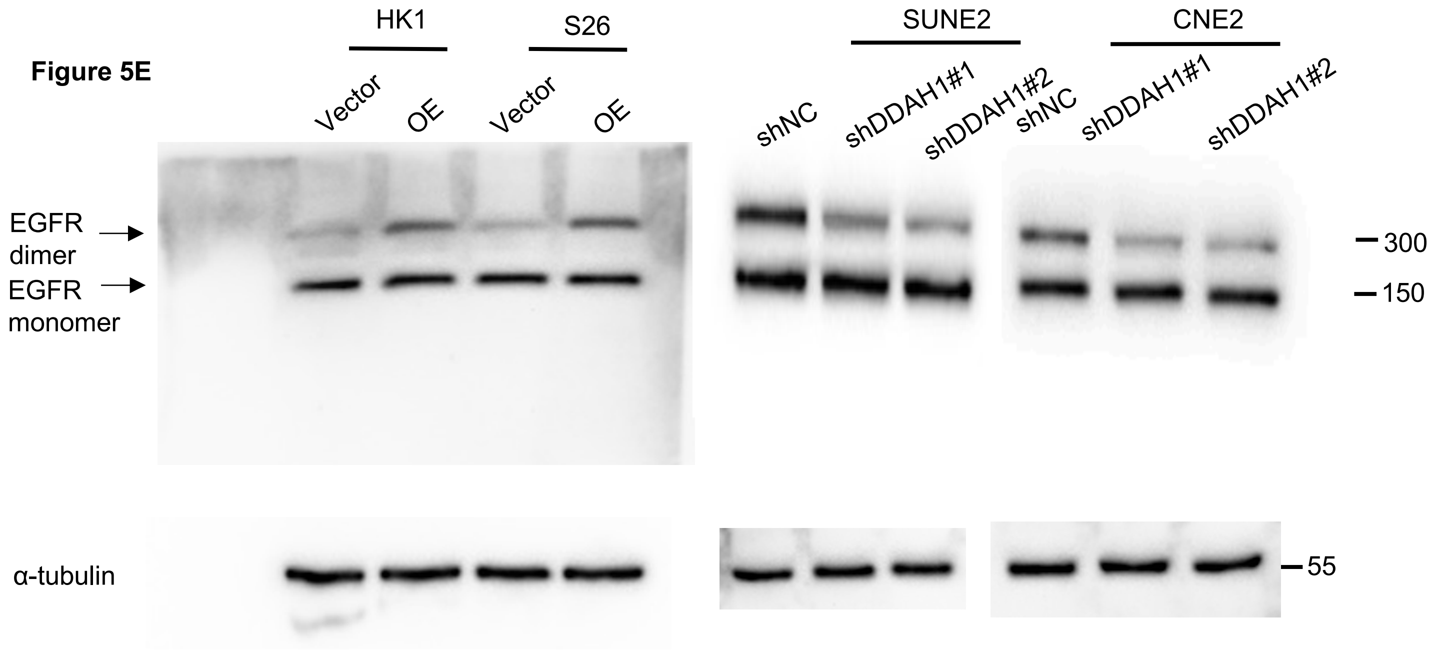


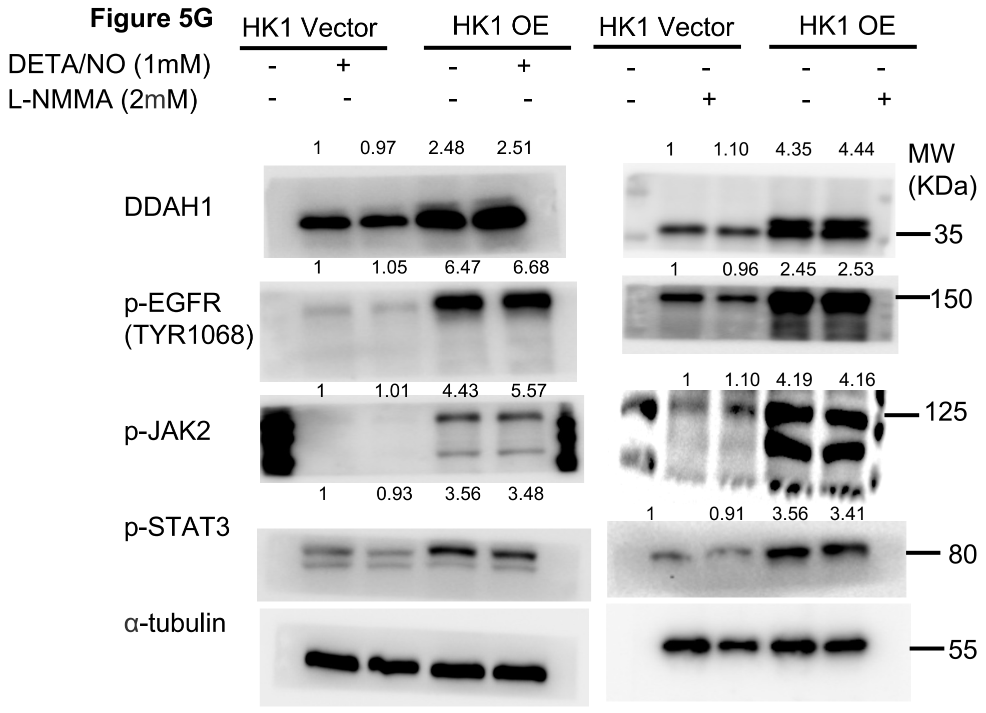


**Supplementary Figure S10.**
Unprocessed blots for Figure 5A, Figure 5B, Figure 5E and Figure 5G in the manuscript.

Supplementary Figure S11 for unprocessed blots

**Figure 6B**

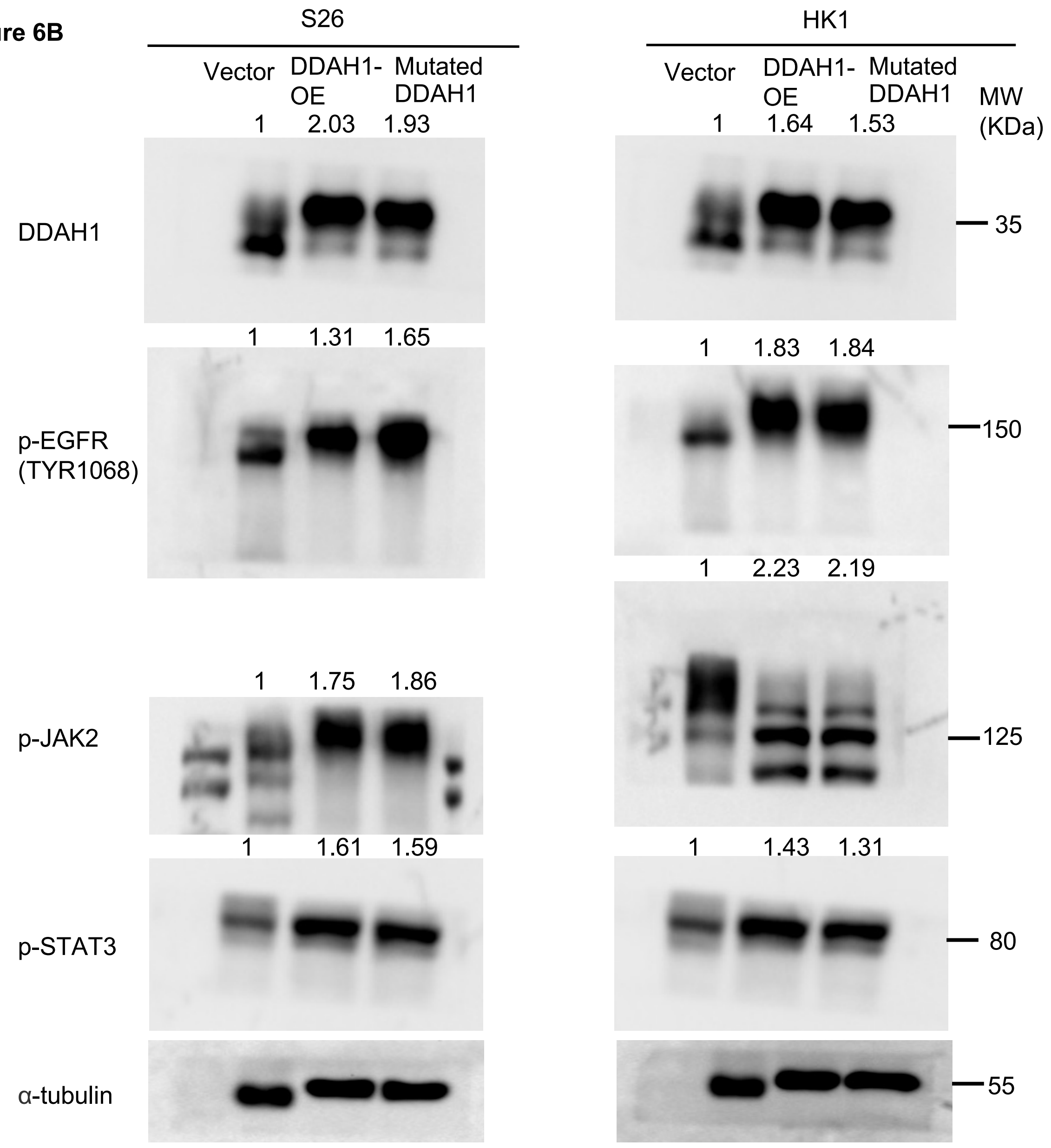


**Figure 7A**

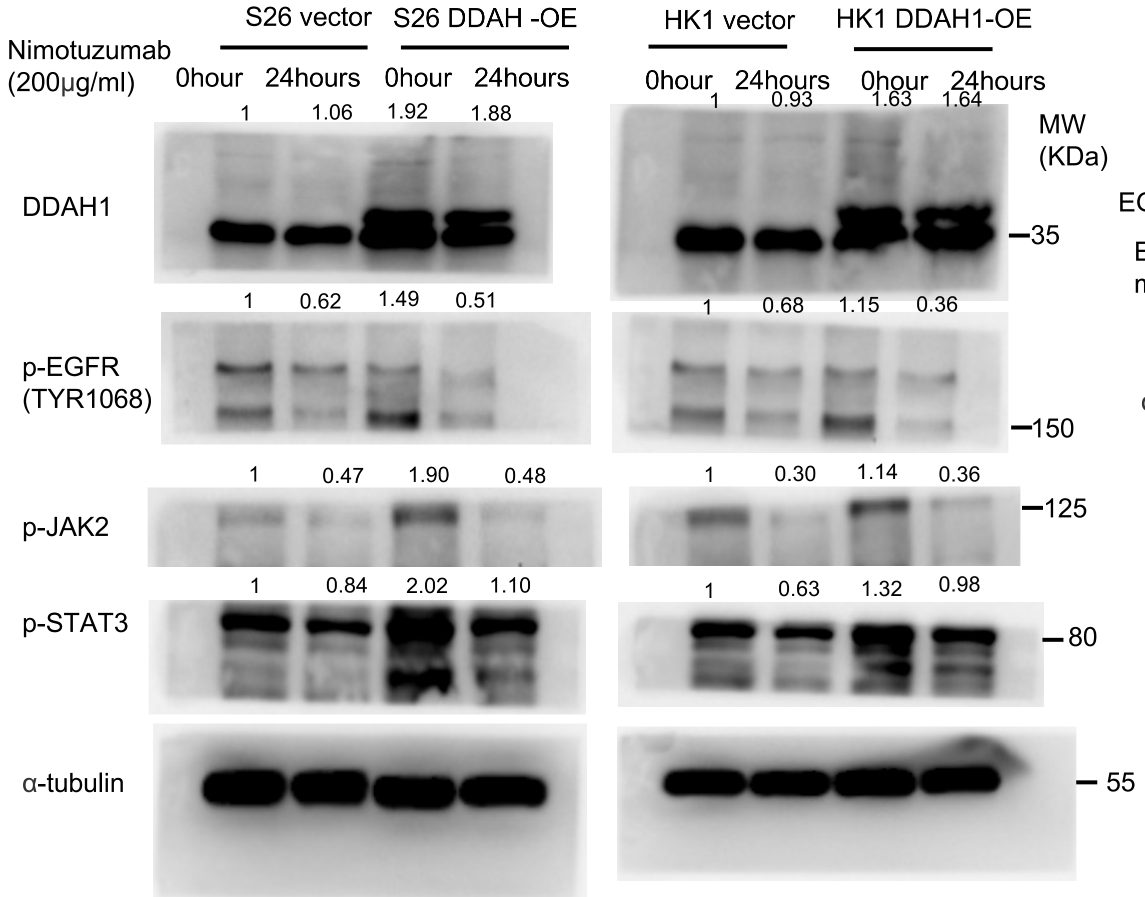


**Figure 7C**

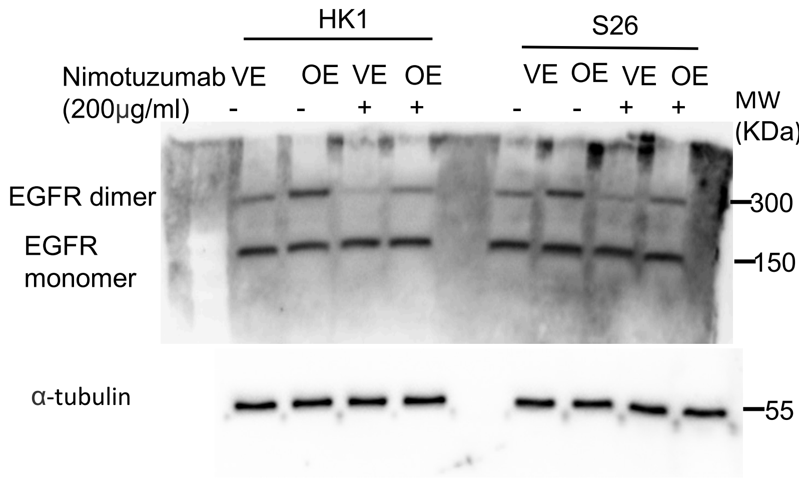


**Figure 7B**

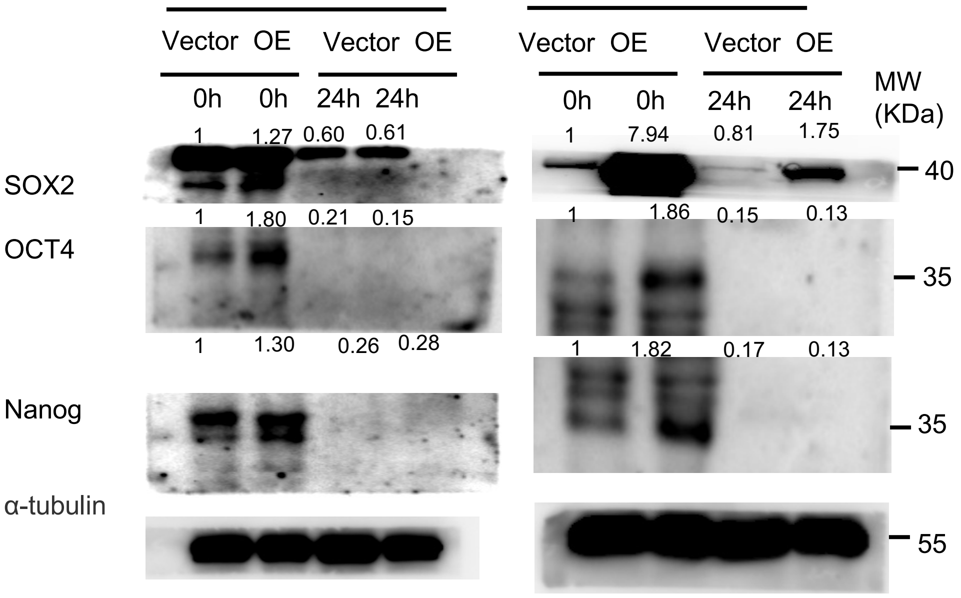


**Supplementary Figure S11.** Unprocessed blots for Figure 6B, Figure 7A, Figure 7B and Figure 7C in the manuscript.

Supplementary Figure S12 for unprocessed blots

**Figure S1A**

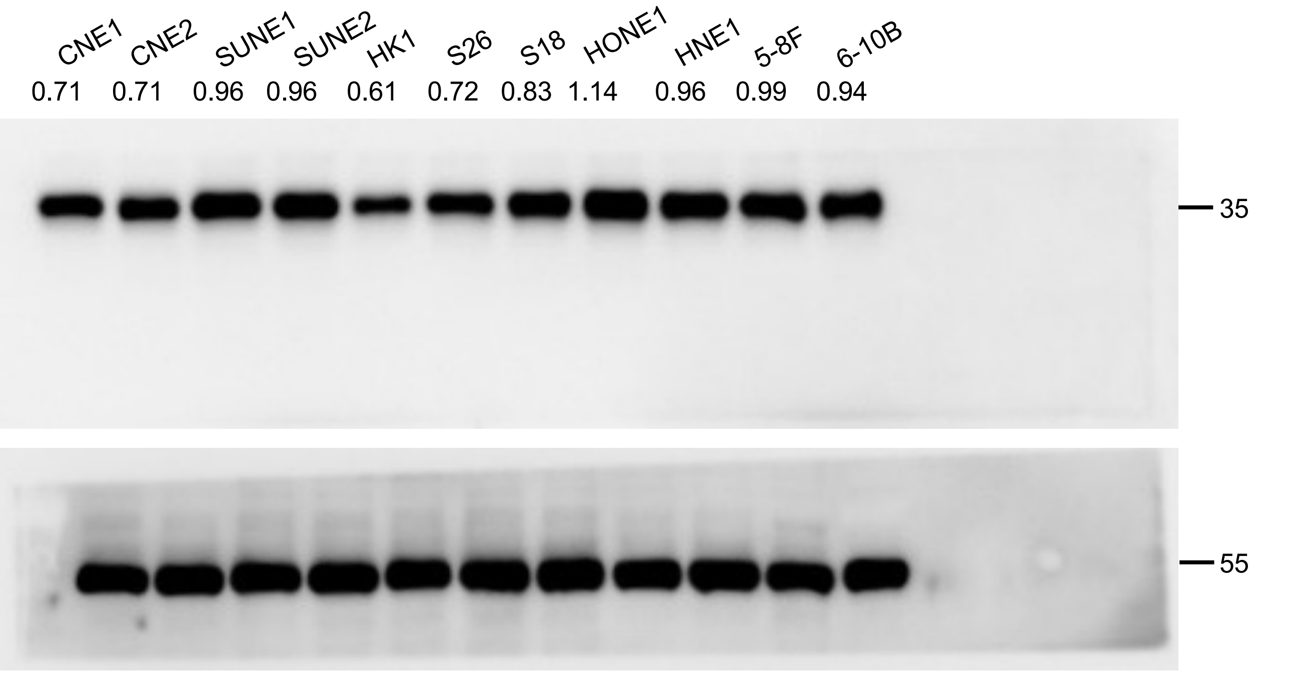


**Figure S6A**

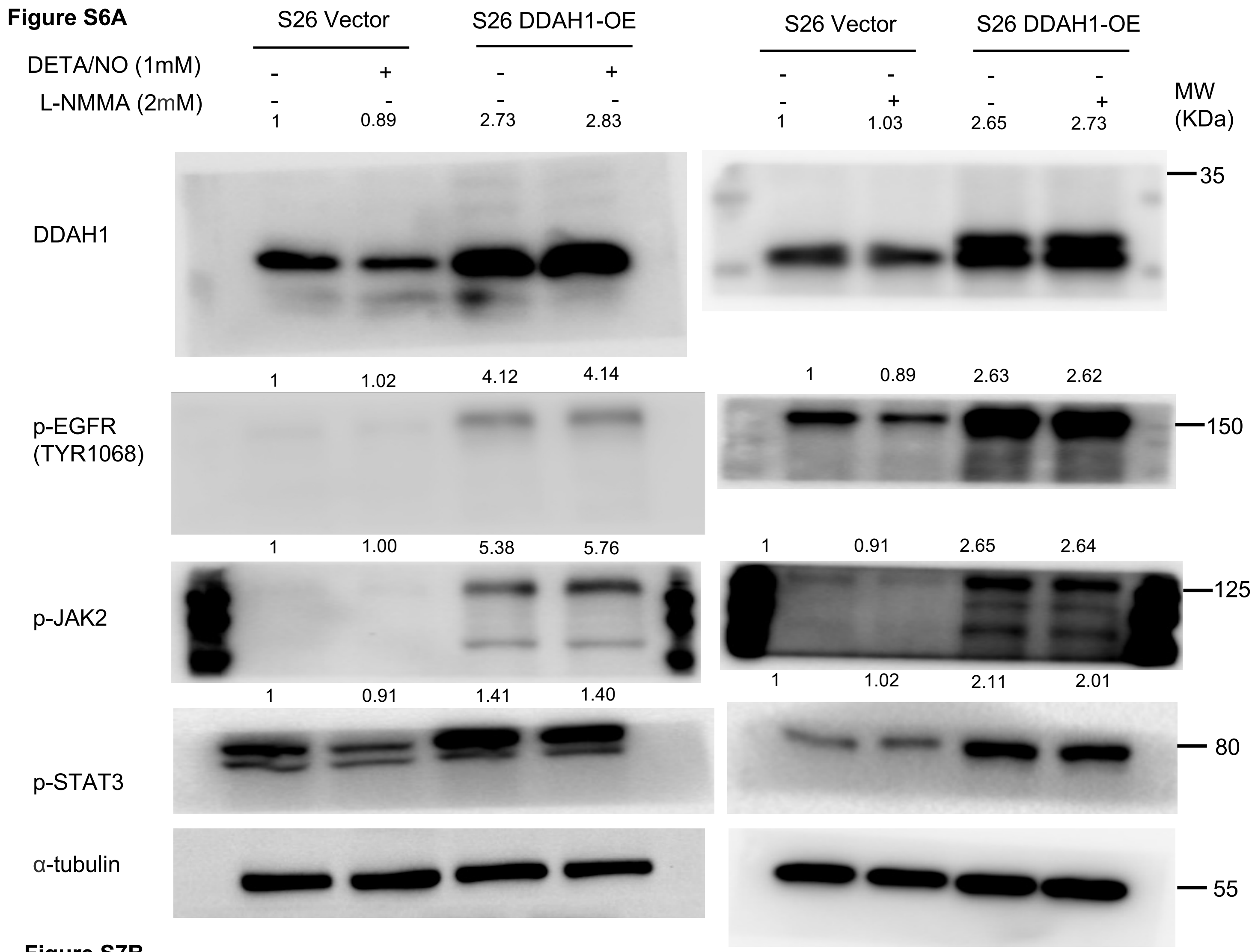


**Figure S7B**

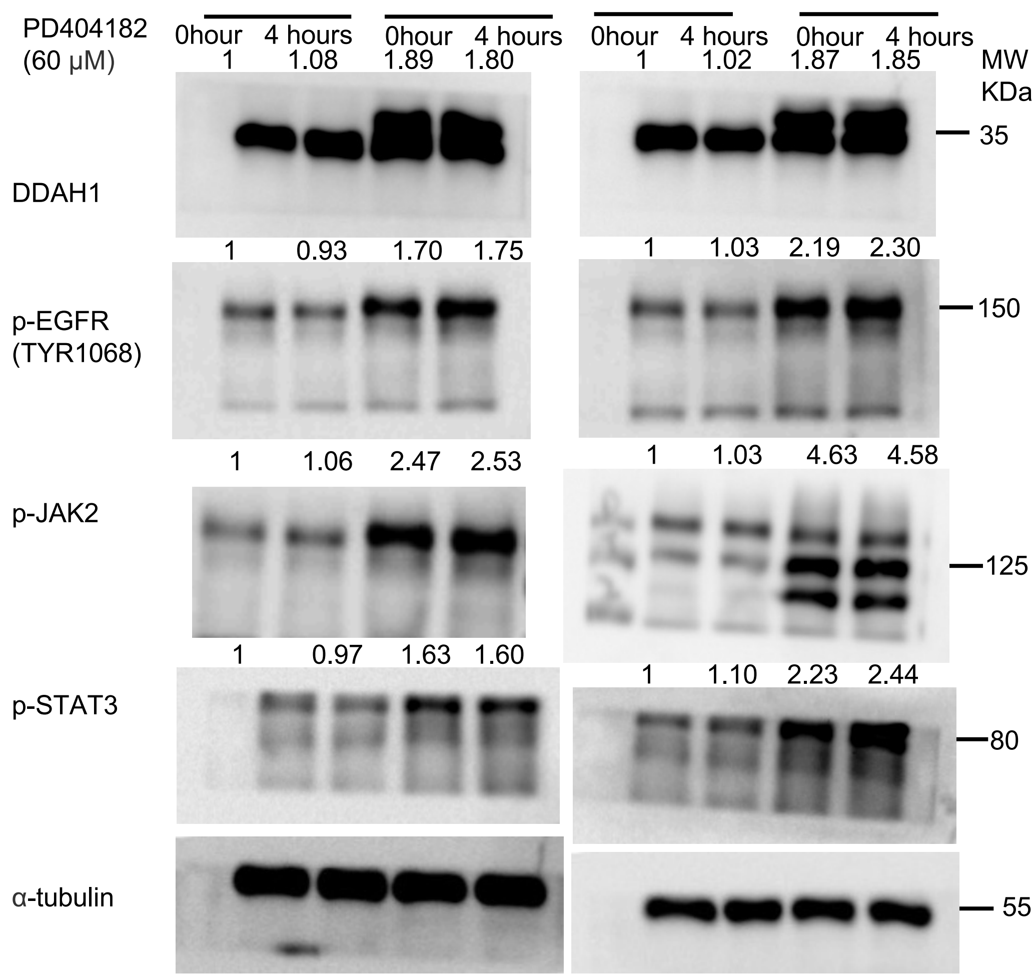


**Supplementary Figure S12.** Unprocessed blots for Figure S1A, Figure S6A and Figure S7B in the manuscript.
